# Regulation of Glandular Size and Phytoalexin Biosynthesis by a Negative Feedback Loop in Cotton

**DOI:** 10.1002/advs.202403059

**Published:** 2024-06-05

**Authors:** Wen‐Kai Wu, Gui‐Bin Nie, Jia‐Ling Lin, Jia‐Fa Huang, Xiao‐Xiang Guo, Mei Chen, Xin Fang, Ying‐Bo Mao, Yan Li, Ling‐Jian Wang, Xiao‐Yuan Tao, Yiqun Gao, Zuo‐Ren Yang, Jin‐Quan Huang

**Affiliations:** ^1^ National Key Laboratory of Plant Molecular Genetics CAS Center for Excellence in Molecular Plant Sciences Shanghai Institute of Plant Physiology and Ecology Chinese Academy of Sciences Shanghai 200032 China; ^2^ University of Chinese Academy of Sciences Beijing 100049 China; ^3^ School of Life Science and Technology ShanghaiTech University Shanghai 200031 China; ^4^ State Key Laboratory of Phytochemistry and Plant Resources in West China Kunming Institute of Botany Chinese Academy of Sciences Kunming 650204 P. R. China; ^5^ Shandong Laboratory of Yantai Drug Discovery Bohai Rim Advanced Research Institute for Drug Discovery Yantai Shandong 264117 China; ^6^ State Key Laboratory of Drug Research Shanghai Institute of Materia Medica Chinese Academy of Sciences Shanghai 201203 China; ^7^ Xianghu Laboratory Hangzhou 311231 China; ^8^ Department of Plant and Crop Science, School of Biosciences, Sutton Bonington campus University of Nottingham Nottingham LE12 5RD United Kingdom; ^9^ National Key Laboratory of Cotton Bio‐breeding and Integrated Utilization, Institute of Cotton Research Chinese Academy of Agricultural Sciences Anyang Henan 455000 China; ^10^ Western Agricultural Research Center Chinese Academy of Agricultural Sciences Changji Xinjiang 831100 China

**Keywords:** cotton, jasmonate, pigment gland, VQ domain

## Abstract

Plants have evolved diverse defense mechanisms encompassing physical and chemical barriers. Cotton pigment glands are known for containing various defense metabolites, but the precise regulation of gland size to modulate defense compound levels remains enigmatic. Here, it is discovered that the VQ domain‐containing protein JAVL negatively regulates pigment gland size and the biosynthesis of defense compounds, while the MYC2‐like transcription factor GoPGF has the opposite effect. Notably, GoPGF directly activates the expression of *JAVL*, whereas JAVL suppresses *GoPGF* transcription, establishing a negative feedback loop that maintains the expression homeostasis between *GoPGF* and *JAVL*. Furthermore, it is observed that JAVL negatively regulates jasmonate levels by inhibiting the expression of jasmonate biosynthetic genes and interacting with GoPGF to attenuate its activation effects, thereby maintaining homeostatic regulation of jasmonate levels. The increased expression ratio of GoPGF to JAVL leads to enlarged pigment glands and elevated jasmonates and defense compounds, enhancing insect and pathogen resistance in cotton. These findings unveil a new mechanism for regulating gland size and secondary metabolites biosynthesis, providing innovative strategies for strengthening plant defense.

## Graphical abstract

This study elucidates a negative feedback loop model involving a VQ‐domain containing protein and a MYC2‐like protein, whereby the ratio of their mRNA and protein contents regulates the size of pigment glands and the biosynthesis of secondary metabolites in cotton. These discoveries unveil a novel mechanism and present innovative strategies for enhancing plant defense, paving the avenue for the improvement of crops.

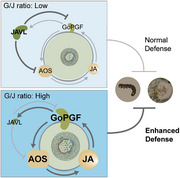

## 1 Introduction

To counteract herbivores and pathogens, plants have evolved various defense mechanisms, primarily encompassing the presence of defense chemicals within secretory structures.^[^ ^1^, ^2^, ^3^ ^]^ In cotton plants, these structures, known as pigment glands, originate from a cluster of gland primordium cells located beneath the epidermis, with the degradation of internal cells leading to the formation of cavities.^[^ ^4^ ^]^ These pigment glands are widely distributed across various aerial organs such as stems, leaves, flowers, and seeds. They serve as repositories for a wide range of secondary metabolites, including volatile terpenes and non‐volatile terpenoids, which confer the plants with distinctive odors and serve as phytoalexins, providing defense against pathogens and insect herbivores.^[^ ^5^, ^6^, ^7^, ^8^ ^]^

Genetic investigations have elucidated the intricate mechanisms underlying the formation of cotton pigment glands, implicating a complex interplay of regulatory factors. The dominant glandless gene *GoPGF* (Gossypium Pigment Gland Formation), also referred to as *Gl2^e^*, was identified in *Gossypium barbadense*, which leads to a glandless phenotype in cotton when silenced. RNA‐seq analysis has identified three cotton *CGFs* (Cotton Gland Formation genes): *CGF1*, *CGF2* and *CGF3*.^[^ ^9^ ^]^ Overexpression of *CGF3* significantly increased terpenoids in tissue culture cells, whereas *CGF2* had less effect on gland density. Silencing *CGF1* and *CGF3* resulted in a significant decrease in gland numbers. Additionally, the gene *GoSPGF* (Gossypium Stem Pigment Gland Forming Gene) regulates gland formation on cotton stalks.^[^ ^10^ ^]^ The MYB transcription factor *CGP1* (Cotton Gland Pigmentation 1) controls gland pigmentation by forming heterodimers in the nucleus through interactions with *GoPGF*.^[^ ^11^ ^]^ Additionally, *JUB1* (*JUNGBRUNNEN 1*), a positive regulator that regulates pigment gland development and gossypol accumulation, potentially operates downstream of *GoPGF* to exert its regulatory influence.^[^ ^12^ ^]^

Despite the identification of several positive regulators associated with pigment gland development,^[^ ^9^, ^10^, ^11^, ^12^, ^13^, ^14^, ^15^ ^]^ the precise molecular mechanisms regulating the pigment gland size remain elusive. Further investigations are needed to unravel the mechanism and develop new strategies for enhancing the production of defense compounds in cotton, thereby boosting its resistance against insects and diseases. In this study, we have unveiled a new intricate regulatory network that governs pigment gland size and the biosynthesis of defense compounds in cotton. We have elucidated a novel negative feedback loop between GoPGF and JAVL, which maintains the balance of jasmonate concentrations and the biosynthesis of secondary metabolites, ultimately influencing the plant's resilience against pathogens and insects. Our study not only advances our comprehension of plant defense mechanisms but also presents innovative strategies for enhancing agricultural productivity and bolstering crop resilience in the face of environmental challenges.

## 2 Results and Discussion

### 2.1 JAVL and GoPGF Exhibit Antagonistical Effects on the Regulation of Pigment Gland Size in Cotton

**Figure 1:**
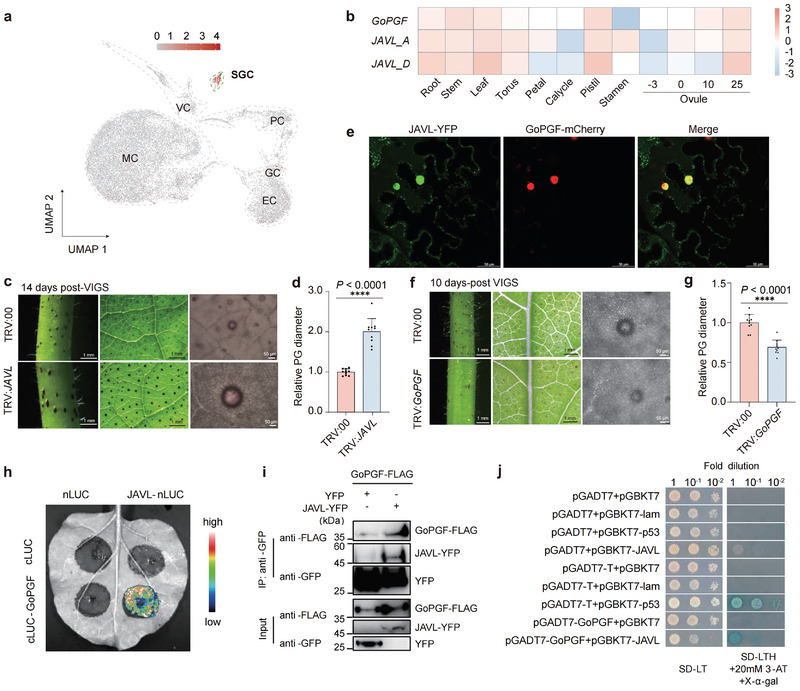
JAVL and GoPGF antagonistically regulate the size of pigment glands in cotton. a) Expression levels of *JAVL* in cotton leaves. Colors in UMAP plot represent scaled expression levels of *JAVL* in individual cells. MC, mesophyll cell; EC, epidermal cell; VC, vascular cell; GC, guard cell; PC, proliferating cell; SGC, secretory gland cell. UMAP, uniform manifold approximation and projection. b) Heatmap showing the expression of *GoPGF*, *JAVL_A* and *JAVL_D* in different tissues and in ovules at different days post anthesis of cotton. Relative expression levels were calculated based on fragments per kilobase per million reads (FPKM) values. c) Phenotypes after *JAVL* VIGS in stems, second true leaves and the single pigment gland at 14 days post‐VIGS treatment compared to the control. TRV:00, empty vector control cotton plants; TRV:*JAVL*, *JAVL*‐VIGS cotton plants. For the phenotypes in stems and second true leaves, scale bars, 1 mm. For the phenotypes in the single pigment gland, scale bars, 50 µm. d) Relative pigment gland (PG) diameter in second true leaves of cotton seedlings after *JAVL* VIGS compared with the control. (mean ± s.d., n = 10, ^****^ *p* < 0.0001, Student' s *t*‐test). e) Confocal microscopy of *N. benthamiana* leaf epidermal cells transiently co‐expressing JAVL‐YFP and GoPGF‐mCherry. Scale bars, 50 µm. f) Phenotypes after *GoPGF* VIGS in stems, first true leaves and single pigment gland at 10 days post‐VIGS treatment. TRV:*GoPGF*, *GoPGF*‐silenced cotton plants. For the phenotypes in stems and first true leaves, scale bars, 1 mm. For the phenotypes in single pigment gland, scale bars, 50 µm. g) Relative pigment gland (PG) diameter in first true leaves of cotton seedlings after *GoPGF* silencing compared with the control (TRV:00). (mean ± s.d., n = 10, ^****^ *p* < 0.0001, Student' s *t*‐test). h) JAVL interacts with GoPGF in vivo represented by bimolecular luciferase complementation assay. The blue‐red gradient indicates the strength of the interactions. i) Co‐IP analysis of the interaction between JAVL and GoPGF in cotyledons of *G.hirsutum*. j) Interactions validation of JAVL and GoPGF by Y2H. For Figure 1d,g, the value of the empty *N. benthamiana* rattle virus vector control (TRV:00) was set to 1.

To identify genes governing pigment gland size, we conducted an in‐depth analysis of our latest single‐cell RNA sequencing data,^[^ ^16^ ^]^ in conjunction with RNA‐seq datasets derived from both glandular and non‐glandular cotton cultivars.^[^ ^17^ ^]^ This comprehensive examination revealed a pair of homoeologous genes (*Gh_A12G0442*/*Gh_D12G0445*), each encoding a VQ domain‐containing protein characterized by a conserved and single short FxxhVQxhTG amino acid sequence motif (Figure S1a, Supporting Information). These two genes were specifically expressed in secretory gland cells and displayed high expression levels in the leaves and ovules of glandular cotton, but were almost undetectable in the corresponding tissues of non‐glandular cotton (**Figure** **1**a; Figure S1b–d, Supporting Information). Furthermore, these genes shared a similar expression pattern with the glandular positive regulator, the MYC2‐like transcription factor *GoPGF*,^[^ ^13^ ^]^ which was highly expressed in stems, leaves, pistils, and ovules at the late stage of ovule development (Figure 1b). Virus‐induced gene silencing (VIGS) of these genes resulted in a conspicuous “big gland” phenotype in the newly emerged leaves and stems compared to the control plants, with the diameter of these enlarged glands approximately doubled (Figure 1c,d; Figure S2a, Supporting Information). The VIGS plants exhibited a notable reduction in gland numbers, coupled with an increase in the relative glandular area per unit leaf size (Figure S2b–d, Supporting Information). These findings demonstrate that these gland‐specific homoeologous genes play a negative regulatory role in modulating pigment gland size in cotton. In comparison to functionally characterized VQ domain‐containing genes, we found that these homoeologous genes exhibit the highest sequence similarity to *JAV1* (Figure S3, Supporting Information), a well‐established negative regulator of jasmonates biosynthesis in *Arabidopsis thaliana*.^[^ ^18^, ^19^ ^]^ Notably, while the *jav1* mutant in *A. thaliana* did not show any visible phenotypes,^[^ ^19^ ^]^ VIGS of these homoeologous genes in cotton resulted in phenotypic changes linked to pigment gland development, indicating significant functional differences between them. From here on, we named these homoeologous genes as jasmonate‐associated VQ motif‐like genes (*JAVL*).

A comparative analysis was performed to assess the sequence similarity between JAVL_A and JAVL_D, unveiling a strikingly high nucleotide similarity of 99% and an amino acid sequence identity of 98% (Figure S4, Supporting Information). Consequently, we hypothesize that JAVL_A and JAVL_D may possess similar function. This phenomenon is a common occurrence in upland cotton, illustrated by enzymatic genes involved in gossypol biosynthesis, such as the P450 genes *CYP706B1* (*Gh_A03G2006*/*Gh_D03G1513*) and *CYP71BE79* (*Gh_A09G2498*/*Gh_D09G0472*), the specialized glyoxalase *SPG* (*Gh_A03G0248*/*Gh_D03G1322*), and the dioxygenase *2‐ODD‐1* (*Gh_D13G2157*/*Gh_A13G2343*).^[^ ^20^, ^21^ ^]^ Despite our assumption that the function of JAVL_A and JAVL_D are equivalent, JAVL_A was nevertheless prioritized as the primary subject for further investigations. To investigate the subcellular localization of JAVL, we transiently expressed JAVL‐YFP in *Nicotiana benthamiana* leaves and the nuclear‐localized GoPGF‐mCherry was used as a positive control.^[^ ^13^ ^]^ In contrast to the nuclear localization of GoPGF, JAVL‐YFP was predominantly observed in the cytoplasm and nucleus (Figure 1e).

To further elucidate the in vivo function of JAVL, we generated *JAVL‐*knockout cotton plants using clustered regularly interspaced short palindromic repeats‐CRISPR‐associated protein 9 (CRISPR‐Cas9) genome editing. Two guide RNAs (sgRNA) were designed to target specific sequences within the single exon of the *JAVL* gene (Figure S5a, Supporting Information), enabling simultaneous targeting of both *JAVL_A* and *JAVL_D*. Subsequently, we generated two distinct knockout alleles of the *JAVL* gene, designated as CR‐*JAVL*#1 and CR‐*JAVL*#2, each characterized by identical 2 bp deletions within the *JAVL_A* sequence. CR‐*JAVL*#1 underwent a 1 bp insertion event within the single exon of *JAVL*_D, while CR‐*JAVL*#2 encountered a 4 bp deletion within the same exon of *JAVL_D*. Both CR‐*JAVL*#1 and CR‐*JAVL*#2 resulted in frameshift mutations, leading to the premature termination of the JAVL protein (Figure S5a, Supporting Information). Remarkably, cotton leaves from *JAVL*‐knockout plants exhibited a significantly pronounced phenotype compared to VIGS plants, with the diameter of the pigment gland increasing to approximately three times that of the wild type (Figure S5b,c, Supporting Information), underscoring the substantial role of JAVL in gland size regulation.

Previous studies have demonstrated that the suppression of *GoPGF* led to the disappearance of glandular structures along with the elimination of associated defense compounds within them.^[^ ^13^ ^]^ Notably, we observed a marked reduction in gland size and a decrease in glandular diameter within the first true leaves of *GoPGF*‐silenced seedlings at 10 days post‐VIGS (Figure 1f,g), indicating a sequential process where the glands initially undergo a reduction in size before ultimately disappearing. Considering JAVL as a negative regulator of pigment gland size, in contrast to GoPGF's role as a positive modulator, we hypothesize the potential existence of competitive interactions between GoPGF and JAVL. Therefore, we next intend to investigate the possibility of JAVL interacting with GoPGF to attenuate its function. A split luciferase complementation (SLC) assay, co‐immunoprecipitation (Co‐IP) and yeast two‐hybrid (Y2H) assays demonstrated a specific interaction between JAVL and GoPGF (Figure 1h–j). These findings suggest a direct interaction between JAVL and GoPGF to control the size of pigment glands.

### 2.2 JAVL and GoPGF Engage in a Negative Feedback to Control the Biosynthesis of Jasmonates

**Figure 2:**
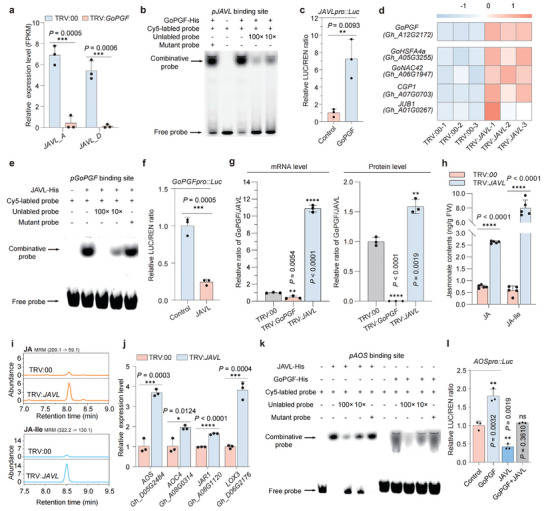
JAVL and GoPGF form a negative feedback loop to modulate jasmonates biosynthesis. a) Relative expression of *JAVL_A* and *JAVL_D* in leaves of TRV:*GoPGF*, compared to the control (TRV:00). TRV:00, empty vector control cotton plants; TRV:*GoPGF*, *GoPGF*‐silenced cotton plants. Relative expression levels were calculated based on FPKM values (mean ± s.d., n = 3, ^***^ *p* < 0.001, Student's *t*‐test). b) GoPGF exhibited binding affinity to the G‐box sequence within the *JAVL* promoter represented by electrophoretic mobility shift assays (EMSA). c) Activation effect of GoPGF on the promoter of *JAVL* was evaluated using dual‐LUC assays (mean ± s.d., n = 3, ^**^ *p* < 0.01, Student's *t*‐test). d) Heatmap illustrating the expression patterns of transcription factors specifically expressed in pigment glands in the leaves of TRV:00 and TRV:*JAVL*. TRV:*JAVL*, *JAVL*‐VIGS cotton plants. Relative expression levels were calculated based on the FPKM values, the color of each square represents the scaled expression levels of the respective genes. *GoPGF* (*Gh_A12G2172*); *GoHSFA4a* (*Gh_A05G3255*); *GoNAC42* (*Gh_A06G1947*); *CGP1*, Cotton Gland Pigmentation 1 (*Gh_A07G0703*); *JUB1*, JUNGBRUNNEN 1 (*Gh_A01G0267*) were selected for analysis. e) EMSA showing the binding affinity of JAVL to the G‐box sequence within the *GoPGF* promoter. f) Dual‐LUC assays illustrating the inhibitory effect of JAVL on *GoPGF* transcription (mean ± s.d., n = 3, ^***^ *p* < 0.001, Student's *t*‐test). g) Relative ratio of GoPGF and JAVL at mRNA (left panel) and protein (right panel) levels in leaves of TRV:*JAVL* and TRV:*GoPGF* compared with the control. (mean ± s.d., n = 3, ^****^ *p* < 0.0001, ^**^ *p* < 0.01, Student's *t*‐test). The value of control group (TRV:00) was set to 1. h) Contents of jasmonates (JA and JA‐Ile) in TRV:*JAVL* leaves compared with the control (TRV:00) (mean ± s.d., n = 5, ^****^ *p* < 0.0001, Student's *t*‐test). i) Extracted ion chromatograms (EIC) of jasmonic acid (JA, *m/z*: 209.1/59.1) and jasmonoyl‐isoleucine (JA‐Ile, *m/z*: 322.2/130.1) extracts from TRV:00 and TRV:*JAVL* leaves. j) Relative expression of *AOS* (*Gh_D05G2484*), *AOC4* (*Gh_A08G0314*), *JAR1* (*Gh_A08G1120*) and *LOX3* (*Gh_D06G2176*) in leaves of TRV:*JAVL* compared with the control (TRV:00) (mean ± s.d., n = 3, ^*^ *p* < 0.05, ^***^ *p* < 0.001, ^****^ *p* < 0.0001, Student's *t*‐test). *AOS*, allene oxide synthase; *AOC4*, Allene oxide cyclase 4; *JAR1*, jasmonoyl amino acid conjugate synthase 1; *LOX*, lipoxygenase 3 were selected for analysis. k) EMSA represents the binding affinity of GoPGF and JAVL to the G‐box sequence within the *AOS* promoter. l) Activation effect of GoPGF and inhibition effect of JAVL on the promoters of *AOS* were evaluated using dual‐LUC assays (mean ± s.d., n = 3, ns *p* ≥ 0.05, ^**^ *p* < 0.01, Student's *t*‐test).

To further investigate the mechanism of JAVL and GoPGF in regulating pigment gland size, we examined the expression level of *JAVL* in *GoPGF*‐silenced cotton plants and found that *JAVL* expression was almost undetectable (**Figure** **2**a; Figure S6, Supporting Information), suggesting that GoPGF may play a role in the activation of *JAVL* expression. Previous studies have suggested that GoPGF can modulate the expression of downstream targets by binding to the G‐box motif within their promoters.^[^ ^16^, ^22^ ^]^ Analysis of the *JAVL_A* and *JAVL_D* promoters revealed the presence of G‐box motifs upstream of the start codons (Figure S7, Supporting Information), and subsequent electrophoretic mobility shift assay (EMSA) confirmed the direct binding of GoPGF to the G‐box motif within the *JAVL* promoter (Figure 2b; Figure S8, Supporting Information). This result was consistent with the chromatin immunoprecipitation sequencing (ChIP‐seq) findings of GoPGF reported by Zhang et al (Figure S9a, Supporting Information).^[^ ^22^ ^]^ Furthermore, a dual‐luciferase reporter (dual‐LUC) system was employed in *N. benthamiana* leaves, where the *JAVL* promoter was fused with the luciferase (*LUC*) gene to generate the *JAVLPro‐LUC* reporter and GoPGF was expressed as an effector protein under the control of 35S promoter. The co‐expression of GoPGF and *JAVLPro‐LUC* led to a remarkable approximately sixfold increase in LUC signal compared to the control group (Figure 2c). These results suggest that GoPGF could activate the expression of *JAVL* by directly binding to the G‐box motif within *JAVL* promoter.

Conversely, the expression of *GoPGF* exhibited a marked increase in *JAVL*‐VIGS plants (Figure 2d; Figure S10, Supporting Information). Additionally, RNA‐seq and qPCR analysis unveiled a significant upregulation of *GoHSFA4a*, *GoNAC42*, *CGP1*, and *JUB1* in *JAVL*‐VIGS plants (Figure 2d; Figure S10, Supporting Information). These genes are recognized downstream targets of GoPGF that positively regulate pigment gland density, pigmentation, or the biosynthesis of defense compounds.^[^ ^11^, ^12^, ^13^, ^16^ ^]^ These findings suggest a potential negative regulation exerted by JAVL on *GoPGF* and its downstream targets. Subsequent validation through EMSA and dual‐LUC assays indicated that JAVL may directly bind to the *GoPGF* promoter, thereby inhibiting its transcriptional activity (Figure 2e,f). These results underscore a negative feedback loop between GoPGF and JAVL in regulating pigment gland development. It is likely that such a negative feedback loop, mediated by JAVL and GoPGF, aids cotton plants in finely tuning their defense mechanisms to cope with turbulent environmental conditions, as negative feedback loops play a crucial role in preventing hyperactivation of specific signaling nodes and maintaining signaling network homeostasis.^[^ ^23^ ^]^

Through qPCR analysis, an unexpected upregulation of *JAVL* gene expression was observed in *JAVL*‐VIGS plants, ≈1.6 times higher than that of the control (Figure S2d, Supporting Information). Conventionally, following VIGS, gene expression levels are generally expected to be downregulated. One plausible explanation is that successful exogenous VIGS leads to targeted degradation of a portion of *JAVL* mRNA, while endogenous upregulation of GoPGF continuously promotes *JAVL* expression, akin to a simultaneous drainage and replenishment process. This possible mechanism strongly supports our model that GoPGF and JAVL could form a negative feedback loop to regulate the homeostasis of gene expression. Moreover, it is noteworthy that after *JAVL*‐VIGS treatment, the expression ratio between *GoPGF* and *JAVL* (*G*/*J* ratio) was significantly elevated (Figure 2g). Conversely, in *GoPGF*‐silenced plants, the *G*/*J* ratio exhibited a marked reduction compared to the control group (Figure 2g), indicating that despite the observed upregulation of *JAVL* expression post‐VIGS, our study compellingly demonstrates the authentic effectiveness of VIGS targeting *JAVL*. In accordance with fluctuations in the *G*/*J* ratio at the mRNA level, our proteomic analysis unveiled that, relative to the control TRV:00 plants (wherein the protein content ratio of GoPGF/JAVL was normalized to 1), the ratio of GoPGF to JAVL protein content elevated by ≈1.6‐fold in *JAVL*‐VIGS plants (Figure 2g). This observation suggests a mitigation of JAVL's repressive function, thereby leading to a diminished inhibitory effect on *GoPGF* expression. This underscores the pivotal role of the GoPGF to JAVL protein content ratio, wherein an elevation in this ratio corresponds to gland enlargement, while a minimal ratio precipitates gland reduction or even disappearance, given the nearly negligible GoPGF/JAVL protein content ratio in TRV:*GoPGF* plants (Figure 2g).

Previous research has demonstrated that JAV1 forms a JAV1‐JAZ8‐WRKY51 (JJW) complex to repress jasmonate biosynthesis in healthy *A. thaliana* plants, maintaining jasmonates at a low level to ensure proper plant growth.^[^ ^18^ ^]^ This prompted us to investigate the impact of *JAVL*‐VIGS treatment on jasmonate levels and the reciprocal influence of jasmonates on *JAVL* expression. In *Arabidopsis*, jasmonates are known to trigger the expression of *JAV1* while facilitating the degradation of the JAV1 protein.^[^ ^13^, ^19^ ^]^ Similarly, our investigations in cotton revealed that methyl jasmonate (MeJA) treatment could induce the expression of *GoPGF* and *JAVL* in cotton (Figure S11, Supporting Information). Moreover, *JAVL‐*VIGS led to a significant increase of jasmonic acid (JA) and jasmonoyl‐isoleucine (JA‐Ile) content compared to control plants (Figure 2h,i). Specifically, the concentration of JA was found to increase approximately fourfold, whereas JA‐Ile levels rose to eightfold compared to those observed in the control plants. And *JAVL*‐VIGS plants exhibited an up‐regulation of jasmonate biosynthetic genes, including lipoxygenase 3 (*LOX3*), allene oxide synthase (*AOS*), allene oxide cyclase 4 (*AOC4*), and jasmonate resistant 1 (*JAR1*) (Figure 2j). Considering the crucial role of *AOS* in the biosynthesis and induction of jasmonates in cotton,^[^ ^24^ ^]^ as well as the repression of *AOS* transcription by JAV1 in *A. thaliana*,^[^ ^18^ ^]^ we next want to explore whether JAVL possesses the ability to repress *AOS* transcription in cotton. EMSA results revealed that JAVL could specifically bind to the G‐box motif of the *AOS* promoter (Figure 2k). Furthermore, our analysis of promoter sequences unveiled G‐box motifs were present in the promoters of several jasmonate biosynthesis genes (Figure S12, Supporting Information), so we cannot exclude the possibility that JAVL might also bind to the promoters of other jasmonate biosynthetic genes. Previous studies have substantiated the existence of a positive feedback loop involving MYC2 and jasmonate biosynthesis genes, including *LOX* and *AOS*, which intricately modulate the precise accumulation of jasmonate levels.^[^ ^25^, ^26^, ^27^ ^]^ In congruence with these established observations, our investigation has unveiled a parallel functional role attributable to GoPGF. Notably, the EMSA result has confirmed that GoPGF could directly bind to the G‐box motif within the *AOS* promoter in cotton (Figure 2k), which is consistent with the ChIP‐seq findings of GoPGF reported by Zhang et al. (Figure S9b, Supporting Information).^[^ ^22^ ^]^ Furthermore, the dual‐LUC assays showed that GoPGF significantly promoted the expression of *AOSPro‐LUC*, whereas JAVL significantly suppressed it compared to the control (Figure 2l). When JAVL and GoPGF were co‐expressed, the *AOSPro‐LUC* expression was comparable to that of the control (Figure 2l), implying the competitive relation between JAVL and GoPGF on the transcriptional regulation of *AOS*. These results suggest that JAVL negatively regulates jasmonate levels by inhibiting the expression of jasmonate biosynthetic genes and interacting with GoPGF to attenuate its activation effect, thereby influencing pigment gland size as a consequence.

### 2.3 Elevated Expression Ratio of *GoPGF* and *JAVL* Contributes to Enhanced Pest and Disease Resistance

**Figure 3:**
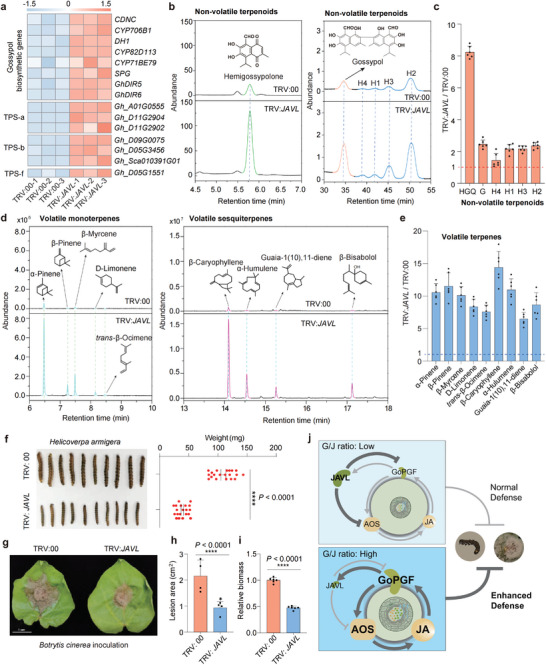
Increased expression ratio of *GoPGF* and *JAVL* enhanced pest and disease resistance. a) Heatmap showing the expression patterns of gossypol and terpene biosynthetic genes in leaves of TRV:*JAVL* and TRV:00. TRV:00, empty vector control cotton plants; TRV:*JAVL*, *JAVL*‐VIGS cotton plants. Relative expression levels were calculated based on FPKM values, the color of each square represents the scaled expression levels of the respective genes. *CDN*, (+)‐δ‐cadinene synthase; *DH1*, alcohol dehydrogenase‐1; *CYP706B1*, *CYP82D113*, *CYP71BE79*, cytochrome P450 monooxygenases; *SPG*, specialized glyoxalase I; *GhDIR5* and *GhDIR6*, dirigent proteins. b) High‐performance liquid chromatography profiles of the extracts derived from cotton leaves subjected to VIGS of *JAVL* in comparison to the control leaves. Peaks corresponding to different terpenoids are indicated by dotted lines. HGQ, hemigossypolone; G, gossypol. H1‐4 are heliocides. c) Statistical analyses of the relative amounts of non‐volatile terpenoids (mean ± s.d., n = 6, Student's *t*‐test). Non‐volatile terpenoids contents in control (TRV:00) plants were normalized to 1. d) Gas chromatography‐mass spectrometry profiles of the extracts derived from cotton leaves subjected to VIGS of *JAVL* in comparison to the control (TRV:00) leaves. The extracted‐ion chromatogram at *m/z* 136 corresponds to the volatile monoterpenes α‐pinene, β‐pinene, β‐myrcene, and *trans*‐β‐ocimene. The extracted‐ion chromatogram at *m/z* 204 illustrates the presence of the volatile sesquiterpenes β‐caryophyllene, α‐humulene, Guaia‐1(10),11‐diene and β‐Bisabolol. Peaks corresponding to different volatile terpenes are indicated by dotted lines. e) Statistical analyses of the relative amounts of volatile terpenes (mean ± s.d., n = 6, Student' s *t*‐test). Volatile terpenes content in control (TRV:00) plants was normalized to 1. f) Growth of *Helicoverpa armigera* larvae fed on leaves of TRV:*JAVL* and TRV:00. The third‐instar larvae were fed on fresh cotton leaves for three days. The weight of the *H. armigera* larvae was measured (mean ± s.d., n = 20, ^****^ *p* < 0.0001, Student's *t*‐test). g) Representative leaves from TRV:00 and TRV:*JAVL* four days after *B. cinerea* inoculation. Scale bars, 1 cm. h–i) Lesion area generated by *B. cinerea* (h) (mean ± s.d., n = 4, ^****^ *p* < 0.0001, Student's *t*‐test) and relative biomass of *B. cinerea* in inoculated cotton leaves (i) were measured four days after inoculation (mean ± s.d., n = 6, ^****^ *p* < 0.0001, Student's *t*‐test). For Figure 3i, the biomass of *B. cinerea* in inoculated control (TRV:00) leaves was set to 1. j) A negative feedback loop model governing pigment gland size and biosynthesis of secondary metabolites for plant defense. Under normal conditions, the G/J ratio typically stays low in both mRNA and protein levels. JAVL functions to suppress the transcription of *GoPGF* and *AOS*, thereby maintaining the low level of jasmonates, the normal size of pigment glands and the basal levels of defense compounds. Following *JAVL*‐VIGS treatment, a substantial increase in the G/J ratio was observed. The inhibitory effect of JAVL reduced significantly, leading to a rapid up‐regulation of *GoPGF* and *AOS* and the accumulation of jasmonates. Consequently, this process contributes to the enlargement of pigment glands and the increased production of defense compounds, thereby enhancing the plant's ability to resist insects and diseases.

In light of the observed increase in gland size in plants subjected to *JAVL*‐VIGS, it became crucial to investigate whether these morphological changes would impact the contents of defense compounds within the pigment glands. RNA‐seq and qPCR analysis of *JAVL*‐VIGS plants revealed a significant upregulation of genes related to gossypol biosynthesis, such as *CDN*, *CYP706B1*, *DH1*, as well as genes encoding secretory dirigent proteins *Gh*DIR5 and *Gh*DIR6, and terpene synthase (TPS) expression, particularly from the TPS‐a (sesquiterpene synthases), TPS‐b (monoterpene synthases), and TPS‐f (have dual activities in monoterpene and sesquiterpene biosynthesis) subfamilies (**Figure** **3**a; Figure S13, Supporting Information). Consistent with the upregulation of these genes, there was a marked increase in non‐volatile terpenoids (hemigossypolone, gossypol, and heliocides 1–4), volatile monoterpenes (α‐pinene, β‐pinene, β‐myrcene, D‐limonene, and *trans*‐β‐ocimene), and sesquiterpenes (including β‐caryophyllene, α‐humulene, guaia‐1(10),11‐diene, and β‐bisabolol) levels in the leaves and stems of *JAVL*‐VIGS plants (Figure 3b–e; Figure S14, Supporting Information). Hemigossypolone showed an approximately sevenfold increase among the non‐volatile terpenoids, while gossypol and heliocides increased by ≈1.5–2‐fold compared to the control (Figure 3c). The changes in volatile terpenes were even more pronounced, with their levels increasing by ≈7–15 times compared to the controls (Figure 3e). Overall, our findings highlight that the modifications in gland size and density induced by *JAVL*‐VIGS have a substantial impact on the biosynthesis and accumulation of both volatile and non‐volatile terpenoids in cotton.

Based on the substantial increase in defense compounds in *JAVL*‐VIGS plants, we hypothesized that the antiherbivore and antimicrobial properties would be significantly enhanced. As expected, cotton bollworm larvae feeding on leaves of *JAVL*‐VIGS plants exhibited markedly growth inhibition (Figure 3f). Furthermore, *JAVL*‐VIGS plants demonstrated enhanced resistance to *Botrytis cinerea*, as indicated by reduced lesion areas and lower pathogen biomass compared to control plants (Figure 3g–i; Figure S15, Supporting Information). These results provide compelling evidence that the manipulation of gland size through *JAVL*‐VIGS in cotton leads to a substantial increase in defense compounds, resulting in enhanced antiherbivore and antimicrobial properties.

## 3 Conclusion

In conclusion, our study has elucidated the antagonistic regulatory roles of GoPGF and JAVL in governing both gland size and the biosynthesis of defense compounds in cotton, establishing a negative feedback loop that orchestrates the balance of their expression levels and jasmonate concentrations. These findings are consistent with previous research in *Arabidopsis*, wherein MYC2/3/4 redundantly regulate wounding‐induced JA accumulation by directly binding to the promoters of JA biosynthesis genes. Additionally, MYC2/3/4 modulate the expression of *JAV1* and *JAM1*, which are key factors controlling JA biosynthesis and catabolism, respectively.^[^ ^28^ ^]^ However, further investigation is warranted to explore whether JAV1 could exert inhibitory effects on *MYC2/3/4* expression in *Arabidopsis*. Moreover, there is compelling evidence indicating the presence of a negative feedback loop involved in the termination of JA responses, as delineated by Liu et al. Specifically, MYC2, in conjunction with the multifunctional Mediator subunit MED25, stimulates the wound‐induced expression of JAMs homologous genes, namely *MYC2‐TARGETED BHLH1* (*MTB1*), *MTB2*, and *MTB3*. Subsequently, MTB proteins disrupt the MED25‐MYC2 interaction and inhibit the DNA binding activity of MYC2, thereby terminating JA‐triggered defense responses through an autoregulatory negative feedback loop.^[^ ^29^ ^]^ Further investigation is imperative to elucidate whether GoPGF and other proteins, in addition to JAV1, exhibit analogous negative feedback loops in cotton pigment glands.

Significantly, we have identified the G/J ratio as a critical modulator of jasmonate levels, where an elevated ratio correlates directly with increased jasmonate concentrations. This increase triggers an enlargement in gland size and an enhanced production of defense compounds, significantly bolstering the plant's resilience against pathogens and insects, as illustrated in Figure 3j. Taken together, our findings unveil a previously unrecognized negative feedback mechanism that regulates pigment gland size and secondary metabolite production in cotton, offering a promising avenue for enhancing agricultural productivity and crop resilience in the future.

## 4 Experimental Section

### Plant Materials and Growth Conditions

Wild‐type upland cotton plants (*Gossypium hirsutum*, variety R15) were cultivated in a greenhouse under controlled conditions with a photoperiod of 16 h of light and 8 h of darkness, maintained at a temperature of 28 °C. *Nicotiana benthamiana* plants were also grown in a greenhouse, with a similar light cycle of 16 h of light and 8 h of darkness, at a temperature of 22 °C.

### Virus‐Induced Gene Silencing (VIGS) Assay

For the VIGS assay, DNA fragments of ≈300–500 base pairs from coding sequences were amplified by PCR using gene‐specific primers and the PCR products were purified and cloned into the *pTRV2* vector. The vectors *pTRV1* and recombinant *pTRV2* vectors were introduced into the *Agrobacterium tumefaciens* strain GV3101 cells by heat shock. The transformed GV3101 cells [OD_600_ = 0.8 in fresh infiltration medium (10 mm MgCl_2_, 150 mm acetosyringone and 10 mm MES; pH 5.8)] carrying assistant *pTRV1* vectors and constructed *pTRV2* vectors at a ratio of 1:1 were infiltrated into the cotyledons of cotton seedlings at ten days old. Two weeks or 10 days after infiltration, the first or second true leaves were collected for further analysis.

### RNA‐Seq

Total RNA was extracted using the RNAprep Pure Plant Plus Kit (DP441, TIANGEN). RNA concentration and purity were measured using NanoDrop 2000 (Thermo Fisher Scientific). RNA integrity was assessed using the RNA Nano 6000 Assay Kit of the Agilent Bioanalyzer 2100 system (Agilent Technologies). A total amount of 1 µg RNA per sample was used as input material for the RNA sample preparations. Sequencing libraries were generated using Hieff NGS Ultima Dual‐mode mRNA Library Prep Kit for Illumina (Yeasen Biotechnology). Briefly, mRNA was purified from total RNA using poly‐T oligo‐attached magnetic beads. First strand cDNA was synthesized and second strand cDNA synthesis was subsequently performed. The remaining overhangs were converted into blunt ends via exonuclease/polymerase activities. After adenylation of 3′ ends of DNA fragments, NEBNext Adaptor with hairpin loop structure was ligated to prepare for hybridization. The library fragments were purified with AMPure XP system (Beckman Coulter). Then 3 µL USER Enzyme (NEB) was used with size‐selected, adaptor‐ligated cDNA at 37 °C for 15 min followed by 5 min at 95 °C before PCR. PCR was then performed with Phusion High‐Fidelity DNA polymerase, Universal PCR primers and Index (X) Primer. Finally, PCR products were purified (AMPure XP system) and library quality was assessed on the Agilent Bioanalyzer 2100 system. The libraries were sequenced on an Illumina NovaSeq platform. The raw reads were further processed with a bioinformatic pipeline tool BMKCloud (www.biocloud.net) online platform. Hisat2 software was used to map with the reference genome, and the StringTie Reference Annotation Based Transcript (RABT) assembly method was used to construct and identify both known and novel transcripts from Hisat2 alignment results.^[^ ^30^, ^31^ ^]^ Quantification of gene expression levels were estimated by fragments per kilobase of transcript per million fragments mapped. Differential expression analysis was performed using the DESeq2.^[^ ^32^ ^]^ Genes with an adjusted P‐value <0.01 & Fold Change ≥ 2 found by DESeq2 were assigned as differentially expressed.

### qPCR Assay

Total RNA was isolated and purified from cotton leaves utilizing an RNAprep Pure Plant Plus Kit (DP441, TIANGEN). After that, ≈1 µg of total RNA was reverse transcribed to cDNA according to the EasyScript One‐Step gDNA removal and cDNA Synthesis SuperMix (AE311‐03, TransGen Biotech). Quantitative PCR with reverse transcription was carried out using a SYBR Green Premix Pro Taq HS qPCR Kit (AG11701, Accurate Biotechnology) to quantify the gene expressions. The cotton histone gene *GhHis* (*Gh_D03G0370*) was set as the housekeeping gene. Three independent biological replicates were used in the experiment. To calculate the expression ratio of *GoPGF* to *JAVL*, the sum of *GoPGF* and *JAVL* expression levels was used as the denominator, while the respective expression levels relative to the internal reference *GhHis* served as the numerator for each gene.

### Protein Extraction and Digestion

For proteomic analysis, plant leaf samples were rapidly frozen in liquid nitrogen. Exactly 200 mg of frozen tissue was accurately weighed and ground into a fine powder in liquid nitrogen with the addition of 40 mg of 0.5% (w/v) insoluble polyvinylpolypyrrolidone. Protein extraction was performed at a ratio of 1:3 (w/v) in homogenization buffer (6 m guanidine hydrochloride, 100 mm Tris‐HCl (pH 8.0), 100 mm EDTA (pH 8.0), 10 mm TCEP. Protease inhibitor cocktail, Roche) followed by sonication at 4 °C for 60 min. The homogenate was then centrifuged at 10 000 g for 20 min at 4 °C. The supernatant was collected, and methanol, chloroform and ddH_2_O were added successively according to the volume ratio of 1:4:1:3 for vortex mixing. The mixture was then centrifuged at 10 000 g for 20 min at 4 °C to achieve phase separation. The upper layer and the interphase were collected for further analysis.

For the upper layer (polypeptide), centrifugal drying at 10 °C under high vacuum was performed, followed by dissolution in 0.1% trifluoroacetic acid, followed by desalting using a C18 column (Waters, WAT054955). The eluate was then centrifugally dried at 10 °C under high vacuum and dissolved in appropriate 0.1% formic acid for instrument analysis.

For the interphase (protein), the pellet was washed three times with 90% acetone, followed by dissolution in 30 µL of 8 m appropriate urea. Protein concentration was determined, and take 100 µg for the following experiment. Then TCEP and chloroacetamide were added and their final concentrations were 10 and 50 mm, respectively, for reduction and alkylation at 37 °C for 30 min. Subsequently, 100 mm ammonium bicarbonate was added to reduce urea concentration to less than 1 m. Trypsin digestion was carried out at a ratio of 1:25 (trypsin: protein) overnight at 37 °C. Following digestion, desalting was performed using a C18 column (Waters, WAT054955), and the eluate was dried by centrifugation. Samples were then dissolved in appropriate 0.1% FA for instrument analysis.

### LC‐MS/MS

For the upper layer, experiments were performed on a Q Exactive HF‐X mass spectrometer that was coupled to Easy nLC (Thermo Fisher Scientific). Samples were reconstituted in 0.1% Formic acid in HPLC‐grade water (0.1% FA). The peptide mixture was loaded onto a the C18‐reversed phase column (25 cm long, 75 µm inner diameter) packed in‐house with RP‐C18 1.9 µm resin in buffer A (0.1% Formic acid in HPLC‐grade water) and separated with a 60 min linear gradient of buffer B (0.1% Formic acid in 80% acetonitrile) at a flow rate of 300 nL min^−1^. MS data was acquired using a data‐dependent top20 method dynamically choosing the most abundant precursor ions from the survey scan (375–1500 *m*/*z*) for HCD fragmentation. Determination of the target value is based on predictive Automatic Gain Control (pAGC). Dynamic exclusion duration was 30 s. Survey scans were acquired at a resolution of 60 000 with AGC target value of 3 × 10^6^ ions or a maximum integration time of 50 ms and resolution for HCD spectra was set to 15 000 with AGC target value of 1 × 10^5^ ions or a maximum integration time of 50 ms. Normalized collision energy was 28 eV. The fixed first *m*/*z* was 200, and the isolation window was 1.6 *m*/*z*.

For the interphase, samples were analyzed using LC‐MS/MS. The system consisted of a timsTOF Pro2 mass spectrometer (Bruker Daltonics) equipped with a nano elute system. A total of 300 ng of sample was injected onto a 25 cm × 75 µm, 1.7 µm, IonOptics analytical column, and separation was achieved using a 60 min gradient at 50 °C. The flow rate was set to 300 nL min^−1^ with A phase as 0.1% FA water and B phase as 0.1% FA acetonitrile. The gradient started at 2% B for 45 min, increased to 22% B within 5 min, further increased to 37% B within 5 min, and finally reached 80% B and held for 5 min. The mass spectrometer operated in diaPASEF mode for DIA data acquisition, scanning from 363.9–1161.9 Da with an isolation window of 14 Da. During PASEF MS/MS scans, collision energy linearly increased from 20 eV (1/K_0_ = 0.6 V.s cm^−2^) to 59 eV (1/K_0_ = 1.6 V.s cm^−2^) with ion mobility.

### Data Analysis of Proteomics

For the upper layer, The MS data were analyzed using Thermo proteome Discoverer2.5.0.400 software. MS data were searched against NBI_Gossypium_hirsutum_v1.1.pep.fas.fa (https://www.cottongen.org/species/Gossypium_hirsutum). An initial search was set at a precursor mass window of 10 ppm. The search followed a No‐enzymatic cleavage rule and allowed maximal three missed cleavage sites and a mass tolerance of 10 ppm for precursor and 0.02 Da for fragment ions. Protein N‐terminal acetylation and methionine oxidation were defined as variable modifications for database searching. The cutoff of global false discovery rate (FDR) for peptide and protein identification was set to 0.01.

For the interphase, Spectronaut18 software was used for DIA data analysis with default parameters (BGS Factory Settings). The sequence database utilized was NBI_Gossypium_hirsutum_v1.1.pep.fas.fa (https://www.cottongen.org/species/Gossypium_hirsutum), and trypsin digestion was specified. Fixed modification was Carbamidomethylation (C) 57.02, and variable modification was Oxidation (M) 15.99. Protein identification criteria were set to Precursor Threshold 1.0% FDR and Protein Threshold 1.0% FDR. A decoy database was generated using the mutated strategy, generating a similar number of amino acid sequences shuffled (minimum 2 amino acids, maximum half of the total peptide length). Spectronaut performed automatic calibration and normalized data using local normalization strategy, utilizing the average peak area of the top 3 peptides with FDR <1.0% for protein group quantitation.

### Histochemistry and Microscopy

Leaves collected from the seedlings after VIGS treatment were cut into 1 cm^2^ pieces and decolorized in 100% ethanol and 80% acetone for 2 h twice orderly. After that, samples were soaked in 10% NaOH solution at 95 °C for 3  h and then rinsed in 1× PBS buffer (pH 7.0) for 30 min three times. The samples were examined and digitally recorded on a microscope.

### Transient Expression in N. benthamiana and G. hirsutum

The coding sequences of *JAVL* and *GoPGF* amplified from total cDNA of cotton leaves were cloned into the *pEAQ* vector harboring the 35S promoter with 6xHis and transformed into *A. tumefaciens* strain GV3101, respectively. The GV3101 cells were cultured in LB medium containing 50 µg mL^−1^ kanamycin, 100 µg mL^−1^ rifampicin and 100 µg mL^−1^ gentamycin, and the suspension was injected into *N. benthamiana* leaves at 4 weeks old or *G. hirsut*um cv. TM‐1 cotyledons at 10 days old. Leaves or cotyledons were detached for further experiments after 72 h for protein expression.

### Subcellular Localization

For subcellular localization analyses, the full‐length coding sequences of *JAVL* and *GoPGF* were recombined into the vector *pCAMBIA1300* to generate *35S::JAVL‐YFP* and *35S::GoPGF‐mCherry* plasmids, respectively. These constructs were transformed into *A. tumefaciens* strain GV3101 and transiently expressed in the *N. benthamiana* leaves with injection method. The YFP (excitation wavelength 515 nm, emission wavelength 527 nm) and mCherry (excitation wavelength 587 nm, emission wavelength 610 nm) fluorescence signals were detected using Leica TSC SP8 STED 3X (Leica) 72 h after injection.

### Split Luciferase Complementation (SLC) Assay

*GoPGF* ligated with *JW772‐35S‐cLUC* and *JAVL* ligated with *JW771‐35S‐nLUC* were constructed and transformed separately into GV3101 (*pSoup‐p19*). The positive colonies were resuspended separately by infiltration medium to OD_600_ ≈1.0, and the *JW771/JAVL‐JW771* strains were mixed with an equal volume of the *JW772/GoPGF‐JW772* strains. After 48 h transient co‐expression, the leaf tissue was infiltrated with 1 mm D‐Luciferin (potassium salt) (APExBIO). The luminescence was detected by Tanon 5200 SF (Tanon).

### Co‐Immunoprecipitation (Co‐IP)

*JAVL* and *GoPGF* were co‐expressed in cotton cotyledons through *pCAMBIA1300‐JAVL‐YFP* and *pEAQ‐GoPGF‐FLAG*, respectively. Total proteins were extracted by IP lysis buffer [50 mm Tris‐HCl, 150 mm NaCl, 20% glycerol, 0.5% NP‐40, 1×PIC (TransGen)] and JAVL fused with YFP tag or control was enriched through GFP‐Trap for 1 h at 4 °C. IP buffer was used to wash GFP‐Trap for five times. Anti‐FLAG western blot was displayed to detect interaction protein fused with FLAG tag.

### Western Blotting

Protein samples were separated in 4–20% ExpressPlus PAGE Gel (GenScript), and transferred to Immobilon‐P PVDF 0.45 µm membrane (Merck). 5% skim milk (BD) anti‐GFP/DDK (FLAG) mAb (1: 5000, ZSGB‐BIO) was the first antibody in 1 × TBST and horseradish peroxidase (HRP) labeled goat anti mouse (1: 10 000, ZSGB‐BIO) was the second antibody. SuperSignal West Femto Maximum Sensitivity Substrate (Thermo Scientific) was added to the PVDF membrane to detect the signal of blotting. The signal was detected by Tanon 5200 SF (Tanon).

### Yeast Two Hybrid (Y2H)

The coding sequences of *JAVL* and *GoPGF* were inserted into *pGBKT7* and *pGADT7* separately. The *JAVL‐pGBKT7* and the *GoPGF‐pGADT7* were co‐transformed into Chemically Competent Cell (Weidibio) AH109, together with control. Growing on the synthetic dextrose minimal medium without Leu and Trp (SD‐LT) two days later, the single colony of co‐transformation AH109 OD_600_ was adjusted to 1.0, then inoculated on synthetic dextrose minimal medium without Leu, Trp and His (0, 20, 40, 60 mm 3‐AT and 15 µg mL^−1^ X‐α‐gal) medium with gradient dilution. Medium culturing AH109 was placed in a 30 °C incubator for 2–3 days.

### Dual‐Luciferase Reporter (dual‐LUC) System Assay

The upstream 2000 bp promoter regions of *JAVL*, *GoPGF* and jasmonate biosynthesis gene *AOS* were amplified from genomic DNA extracted from cotton leaves. These promoters were then ligated to the firefly LUC reporter gene. Subsequently, analysis of the LUC and fluorescence detection were conducted following the protocol of Dual Luciferase Reporter Gene Assay Kit (Yeasen Biotechnology, Shanghai). The LUC activity was normalized to that of the internal control gene *REN*.

### Heterologous Expression of JAVL and GoPGF

JAVL and GoPGF were heterologously expressed in *Escherichia coli* BL21 (DE3) using the *pET32a* vector. Transformants were grown on an LB medium containing 100 µg mL^−1^ ampicillin. A single colony was inoculated in 5 mL of LB medium containing 100 µg mL^−1^ ampicillin and grown overnight at 37 °C, followed by culture in 500 mL LB medium containing 100 µg mL^−1^ ampicillin until OD_600_ ≈0.6, and induced with 250 µm IPTG at 18 °C for 20 h. Recombinant proteins were purified with Ni‐NTA resin (DP101‐02, TransGen Biotech). Protein concentration was determined using the Bradford method.

### Electrophoretic Mobility Shift Assays (EMSAs)

EMSAs were carried out as described previously.^[^ ^33^ ^]^ Cy5‐labeled probes, unlabeled competitive probes and unlabeled mutant probes were synthesized by Sangon (Shanghai, China) and are detailed in Table S1 (Supporting Information). The purified GoPGF and JAVL proteins were incubated with the probes at 30 °C for 30 min and subsequently separated via 5% native PAGE (10 V cm^−1^, 4 °C) in Tris/borate/EDTA buffer. Unlabeled competitive probes and unlabeled mutant probes were used as cold competitors. 100x and 10x indicate the folded excess of the cold competitor relative to that of the labeled probe, while the unlabeled mutant probes did not affect the binding of the Cy5‐labeled probes to the proteins. Fluorescence was detected using an image scanner (FLA‐9000; Fujifilm).

### Quantification of Jasmonates

For jasmonates (JA and JA‐Ile) extraction, frozen leaves (100 mg) were ground into fine powder in liquid nitrogen. Then samples were extracted with 500 µL ethyl acetate twice, followed by ultrasonic extraction for 15 min, drying ethyl acetate out by vacuum concentrator (Eppendorf, Hamburg, Germany) and redissolving in 30% methanol for detection of jasmonates. An Agilent 6475 Triple Quadrupole LC/MS system was used for the quantification of jasmonates. Separation was carried out on an XSelect HSS T3 column (3.0 × 100 mm, 2.5 µm). A gradient elution as follows: 90–90% A, 0–2.0 min; 90–10% A, 2.0–9.0 min; 10–1% A, 9.0–11.0 min; 1–90% A, 11.0–11.1 min; 90‐90% A, 11.1–13.0 min, was run at a flow rate of 0.3 mL min^−1^ with the solvent system comprised solvent A (2 mm ammonium formate in 0.01% formic acid solution) and solvent B (acetonitrile). The mass spectrometer was set to multiple reaction monitoring (MRM) mode using electrospray ionization (ESI) in negative ion mode. The transitions from deprotonated molecules to characteristic product ions were monitored for jasmonic acid (JA) (*m/z*, 209.1/59.1) and jasmonoyl‐isoleucine (JA‐Ile) (*m/z*, 322.2/130.1), using the following parameters: fragmentor voltage (JA, 112 V; JA‐Ile, 159 V); collision energy (JA, 13 V; JA‐Ile, 25 V); cell accelerator voltage (JA, 4 V; JA‐Ile, 4 V). The peak areas corresponding to the identified jasmonates were quantified utilizing calibration curves established with the standards JA and JA‐Ile. Consequently, this methodology enables the determination of absolute concentrations for JA and JA‐Ile. All the experiments were repeated for five biological times to calculate the mean ± s.d.

### Quantification of Gossypol, Hemigossypolone, and Heliocides

Gossypol, hemigossypolone, and heliocides were quantified as previously reported.^[^ ^21^ ^]^ Plant leaves were powdered in liquid N_2_ and treated with 1 mL of extraction buffers per 100 mg (acetonitrile: water: phosphoric acid = 80:20:0.1), after finely mixing, ultrasonic extraction for 15 min and centrifugation for 5 min at 13 400 g. The supernatant was then filtered through 0.22 µm PTFE filters and then injected in Agilent 1260 Infinity II LC System, with a Thermo Scientific Syncronis C18 column (150 × 4.6 mm, 5 µm). The solvent system containing solvent A (EtOH:MeOH:IPA:ACN:H_2_O:EtOAc:DMF:H_3_PO_4_ = 16.7:4.6:12.1:20.2:37.4:3.8:5.1:0.1) was used at a flow rate of 1 mL min^−1^ for 60 min, and the column temperature kept at 40 °C during the procedure. The determination wavelength was 272 nm. Quantification was performed using the Agilent 1260 system and retention time matched standards was used for compound identification.

### Quantification of Volatile Terpenes

Fresh plant tissues (100 mg) were pulverized in liquid N_2_ and subjected to extraction with 1.5 mL hexane in a vortex for 2 min, followed by analysis using GC‐MS (Agilent 6890 Series GC System coupled to an Agilent 5973 Network Mass Selective Detector), with the following program: initial temperature 40 °C (2 min hold), increase to 240 °C at 10 °C min^−1^. The flow rate of the carriage gas (He) was 1 mL min^−1^. Split injection with a 5:2 ratio. The mass spectral data ranging from *m/z* 30 to 550 were recorded.

### Insect Feeding Assay

The third instar larvae of *Helicoverpa armigera* (Keyun Biology) were reared under conditions of 25 °C, photoperiod 14 h light/10 h dark and relative humidity of 70%. These larvae were divided into 20 larvae per group and fed on freshly collected *JAVL*‐VIGS or control cotton lines. When feeding on plants, individual larva was raised in separate containers and transferred to fresh and redundant plants once a day. After 3 days of feeding according to the above feed, the weight was recorded.

### Pathogen Inoculation Assay

Pathogens were inoculated as previously described.^[^ ^34^ ^]^ Spores of *Botrytis cinerea* strain B05.10 were cultured on Potato Dextrose Agar (PDA) medium for 3 days, and then 1 cm^2^ colonies were transplanted to the new PDA medium for further culture for 5 days. The detached leaves of cotton plants after 2 weeks of VIGS infiltration were infected by *B.cinerea*. The lesion area was measured by Image J software.

### Data Analysis and Statistics

All experiments were repeated using at least three biological replicates. Compound structures were generated using ChemDraw 19.0 software. Chromatograms were plotted using Origin 2022. The quantification and statistical analysis of the compounds were performed using GraphPad Prism 8.3.0. All experimental data were expressed as the mean ± s.d., and statistical significance was determined using a two‐tailed unpaired Student's *t*‐test. The significance levels were denoted as ns *p* ≥ 0.05, ^*^ *p* < 0.05, ^**^ *p* < 0.01, ^***^ *p* < 0.001, and ^****^ *p* < 0.0001.

### Gene Editing and Cotton Transformation

The gene knockout mediated by CRISPR‐Cas9 was performed by utilizing an online toolkit (http://crispr.hzau.edu.cn/CRISPR2/) to analyze the genomic DNA sequence of *JAVLs*. To enhance the editing efficiency, two potential target sites (GATCATCTCTTCCTGAGCTA+AGG and GCTGGCGTCAGCATTGAGAA+GGG) were selected and assembled into the *p7N‐Cas9* vector. *G. hirsutum* cv. TM‐1 was stably transformed. Genomic DNA from both wild‐type and transgenic cotton lines was extracted using a DNA extraction kit (Bioteke), followed by PCR amplification of the target genes. The PCR products were then ligated into a TA‐cloning vector (*CB501‐2*, TransGen Biotech) and subjected to Sanger sequencing for further analysis.

### Data Availability

The authors declare that all relevant data supporting the findings of this study are available within the research and its Supporting Information. The RNA‐seq data produced in this study have been deposited to (the National Center for Biotechnology Information) under the accession number PRJNA1081948. Public RNA‐seq data can be obtained from the SRA repository (https://www.ncbi.nlm.nih.gov/sra) under accession PRJNA248163, PRJNA265955 and PRJNA493958. Sequence data and gene ID can be found in the Cottongen database (https://www.cottongen.org). Furthermore, datasets generated and/or analyzed during the current study are available from the corresponding author upon reasonable request.

## Conflict of Interest

The authors declare no conflict of interest.

## Author Contributions

W.‐K.W., G.‐B.N., J.‐L.L., and J.‐F.H. contributed equally to this work. J.‐Q.H. conceived and designed the study. W.‐K.W., G.‐B.N., J.‐L.L., and J.‐F.H. performed most of the experimental work with help from X.‐X.G., M.C., X.F., Y.‐B.M., Y.L., L.‐J.W., X.‐Y.T., Y.‐Q.G., and Z.‐R.Y. discussed the results and provided advice. W.‐K.W., G.‐B.N., and J.‐L.L. analyzed the data. J.‐Q.H., W.‐K.W., G.‐B.N., J.‐L.L., and J.‐F.H. wrote the paper. All authors reviewed and edited the paper.

## Supporting information

Supporting Information

## Data Availability

The data that support the findings of this study are available from the corresponding author upon reasonable request.
