# Supplementary material for: Regulation of Glandular Size and Phytoalexin Biosynthesis by a Negative Feedback Loop in Cotton
Source: Adv Sci (Weinh). 2024 Jun 5;11(30):2403059. doi: 10.1002/advs.202403059 (PMC11321651; doi:10.1002/advs.202403059)
Supplement: Supplementary file 1 — Supporting Information [file ADVS-11-2403059-s001.pdf]

ADVANCED SCIENCE
Open Access

Supporting Information

for *Adv. Sci.*, DOI 10.1002/advs.202403059

Regulation of Glandular Size and Phytoalexin Biosynthesis by a Negative Feedback Loop in Cotton

*Wen-Kai Wu, Gui-Bin Nie, Jia-Ling Lin, Jia-Fa Huang, Xiao-Xiang Guo, Mei Chen, Xin Fang, Ying-Bo Mao, Yan Li, Ling-Jian Wang, Xiao-Yuan Tao, Yiqun Gao, Zuo-Ren Yang and Jin-Quan Huang**

Supporting Information

Regulation of Gland Development and Phytoalexin Biosynthesis by a Negative Feedback Loop in Cotton

Wen-Kai Wu[1,2,11], Gui-Bin Nie[1,2,11], Jia-Ling Lin[1,3,11], Jia-Fa Huang[1,2,11], Xiao-Xiang Guo[1], Mei Chen[1], Xin Fang[4], Ying-Bo Mao[1], Yan Li[5,6], Ling-Jian Wang[1], Xiao-Yuan Tao[7], Yi-Qun Gao[8], Zuo-Ren Yang[9,10] and Jin-Quan Huang[1*]

[1]National Key Laboratory of Plant Molecular Genetics, CAS Center for Excellence in Molecular Plant Sciences, Shanghai Institute of Plant Physiology and Ecology, Chinese Academy of Sciences, Shanghai 200032, China.

[2]University of Chinese Academy of Sciences, Beijing 100049, China

[3]School of Life Science and Technology, ShanghaiTech University, Shanghai 200031, China

[4]State Key Laboratory of Phytochemistry and Plant Resources in West China, Kunming Institute of Botany, Chinese Academy of Sciences, Kunming 650204, P. R. China

[5]Shandong Laboratory of Yantai Drug Discovery, Bohai Rim Advanced Research Institute for Drug Discovery, Yantai 264117, Shandong, China

[6]State Key Laboratory of Drug Research, Shanghai Institute of Materia Medica, Chinese Academy of Sciences, Shanghai 201203, China

[7]Xianghu Laboratory, Hangzhou 311231, China

[8]Future Food Beacon of Excellence & School of Biosciences, University of Nottingham, Sutton Bonington, UK.

[9]National Key Laboratory of Cotton Bio-breeding and Integrated Utilization, Institute of Cotton Research, Chinese Academy of Agricultural Sciences, Anyang 455000, Henan, China

[10]Western Agricultural Research Center, Chinese Academy of Agricultural Sciences, Changji 831100, Xinjiang, China

[11]These authors contributed equally to this article.

[*] Corresponding author. Email: huangjinquan@cemps.ac.cn

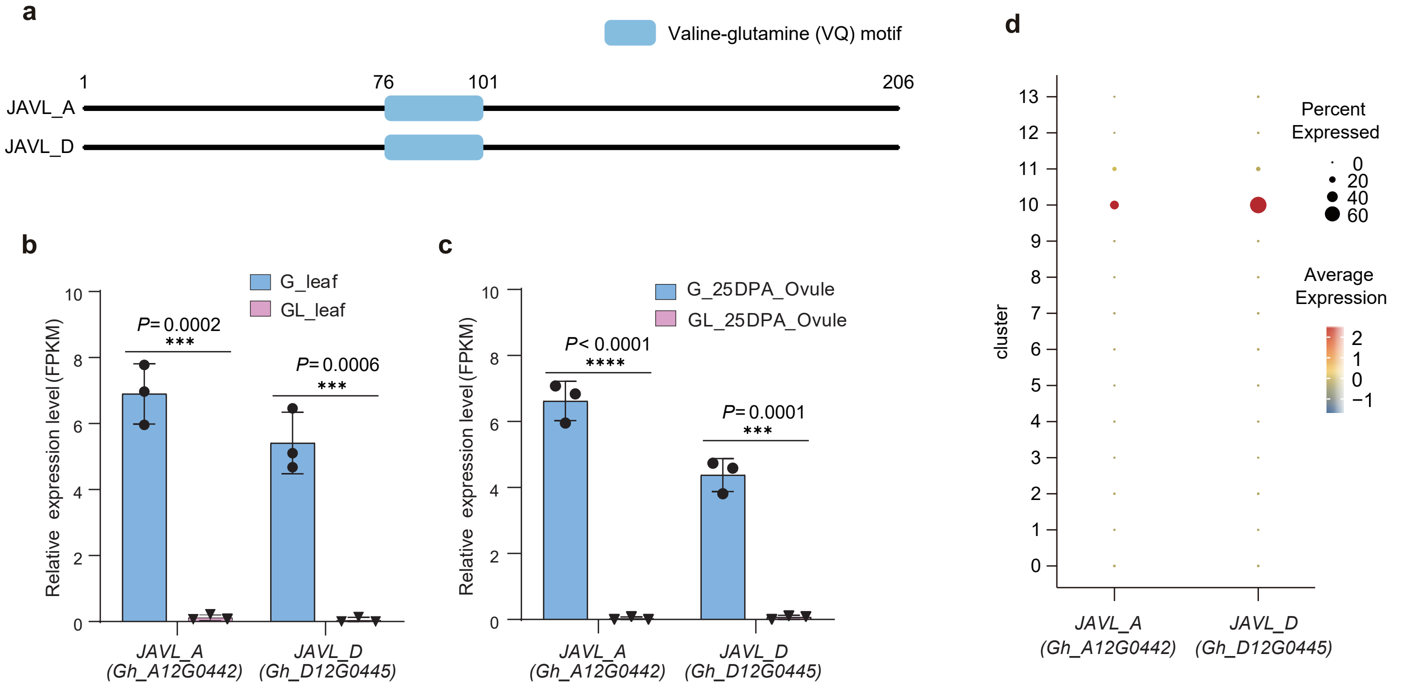

Figure S1. Expression characteristics of *JAVL*. a, Visualization of valine-glutamine (VQ) motif within JAVL_A and JAVL_D. **b-c**, Relative expression of *JAVL_A* and *JAVL_D* in leaves (**b**) and 25-DPA ovules (**c**) of glandular cotton and glandless cotton. G, glandular cotton line; GL, glandless cotton line. DPA, days post anthesis. Relative expression levels were calculated based on FPKM values (mean ± s.d., n = 3, *****P* < 0.0001, Student's *t*-test). **d**, Dot plot visualization of *JAVL_A* and *JAVL_D* in cotton leaves scRNA-Seq data. The size of the dot encodes the expression percentage of cells within a cell type, and the co lor encodes the average expression level. Cluster 0, 1, 3, 4 and 5: mesophyll cells. Cluster 2: epidermal cells. Cluster 6, 9, 12 and 13: vascular cells. Cluster 11: guard cells. Cluster 7 and 8: proliferating cells. Cluster 10: secretory gland cells.

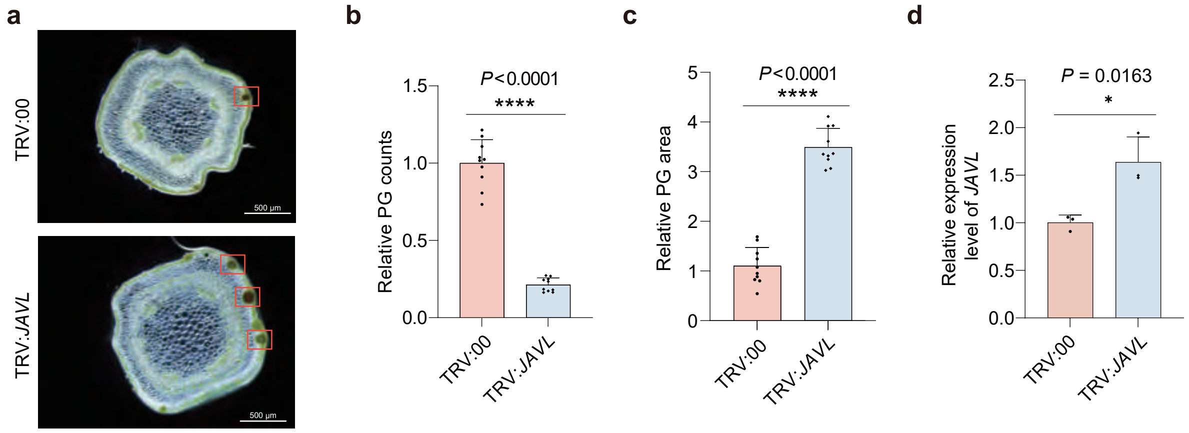

Figure S2. Glandular characterization of *JAVL*-VIGS plants. a, Transverse section of stems of the *JAVL*-VIGS and control group cotton seedlings 14 days post VIGS treatment. Cotton glands were indicated in the red box. Scale bars, 500 μm. **b-c**, Relative pigment gland (PG) counts (**b**) and area (**c**) in second true leaves of cotton seedlings after *JAVL* VIGS compared with the control (mean ± s.d., n = 10, ****P < 0.0001, Student's t-test). Value of the control (TRV:00) was set to 1.TRV:00, empty vector control cotton plants; TRV:-*JAVL*, *JAVL*-VIGS cotton plants. **d**, Relative expression of *JAVL* in *JAVL*-VIGS cotton leaves. Gene expression in control plants (TRV:00) was set as 1 (mean ± s.d., n = 3, *P < 0.05, Student's t-test).

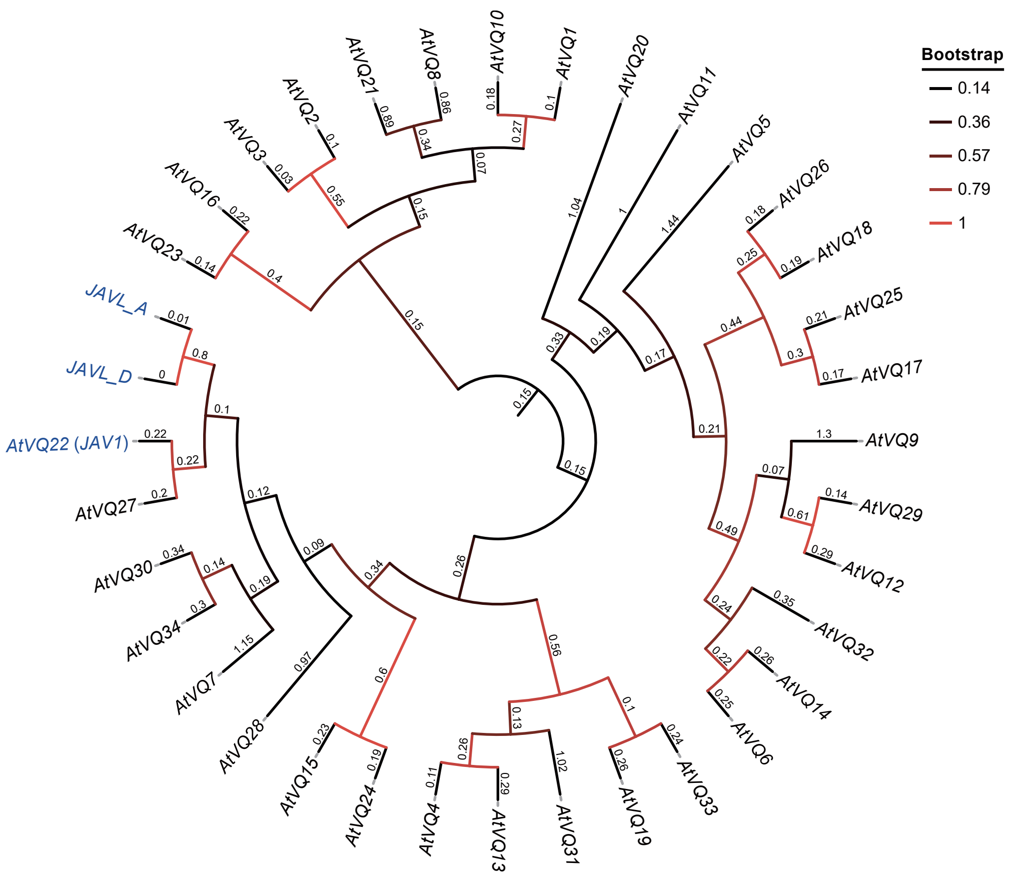

Figure S3. The phylogenetic relationship of *JAVL* with genes encoding VQ motif-containing proteins in *Arabidopsis thaliana*. Maximum likelihood (ML) bootstrap values are shown as gradient red branches. Branch length values are displayed in the major nodes.

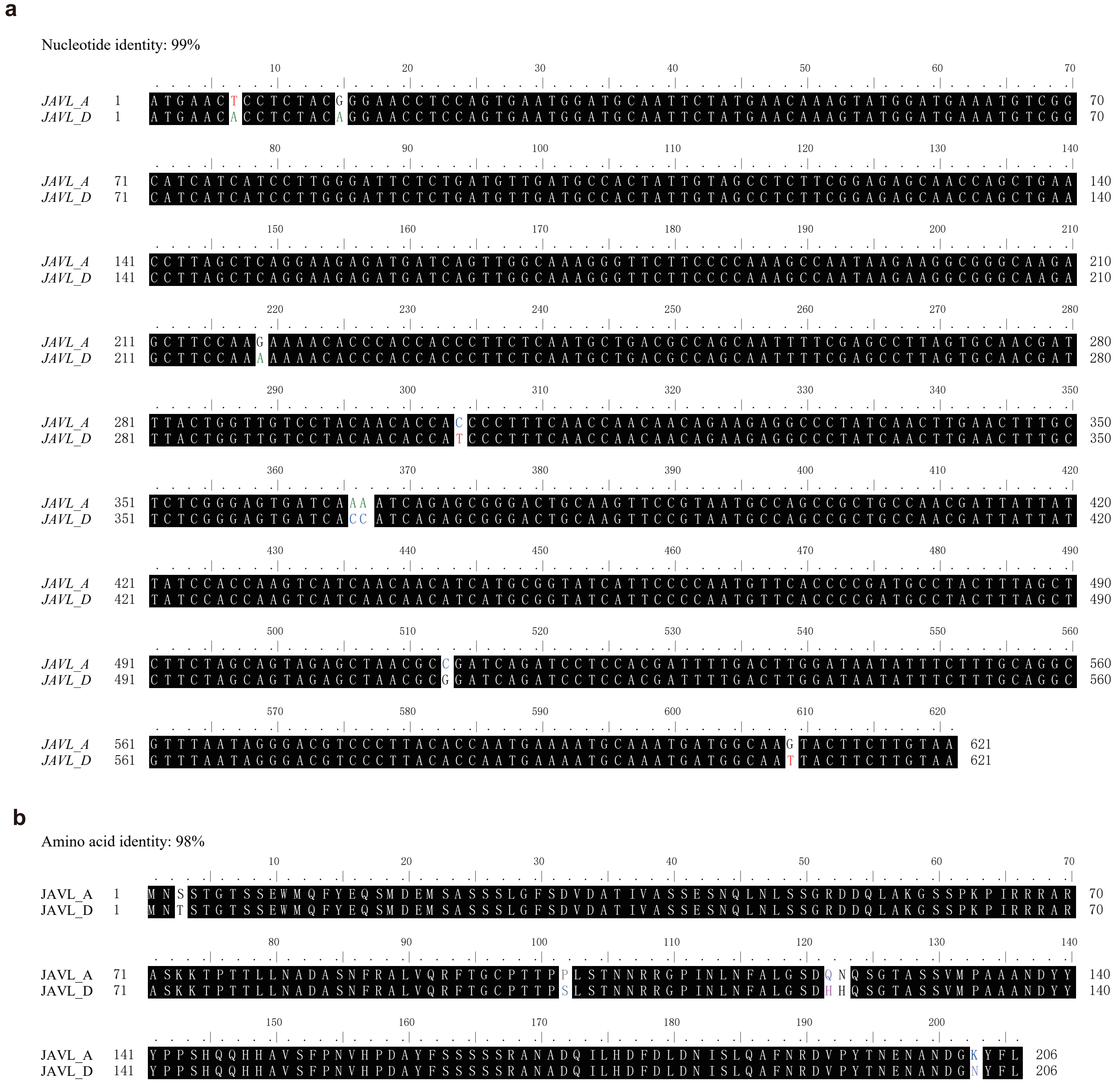

Figure S4. Sequence alignment of JAVL_A and JAVL_D. Coding sequence (**a**) and amino acid sequence (**b**) alignment are shown.

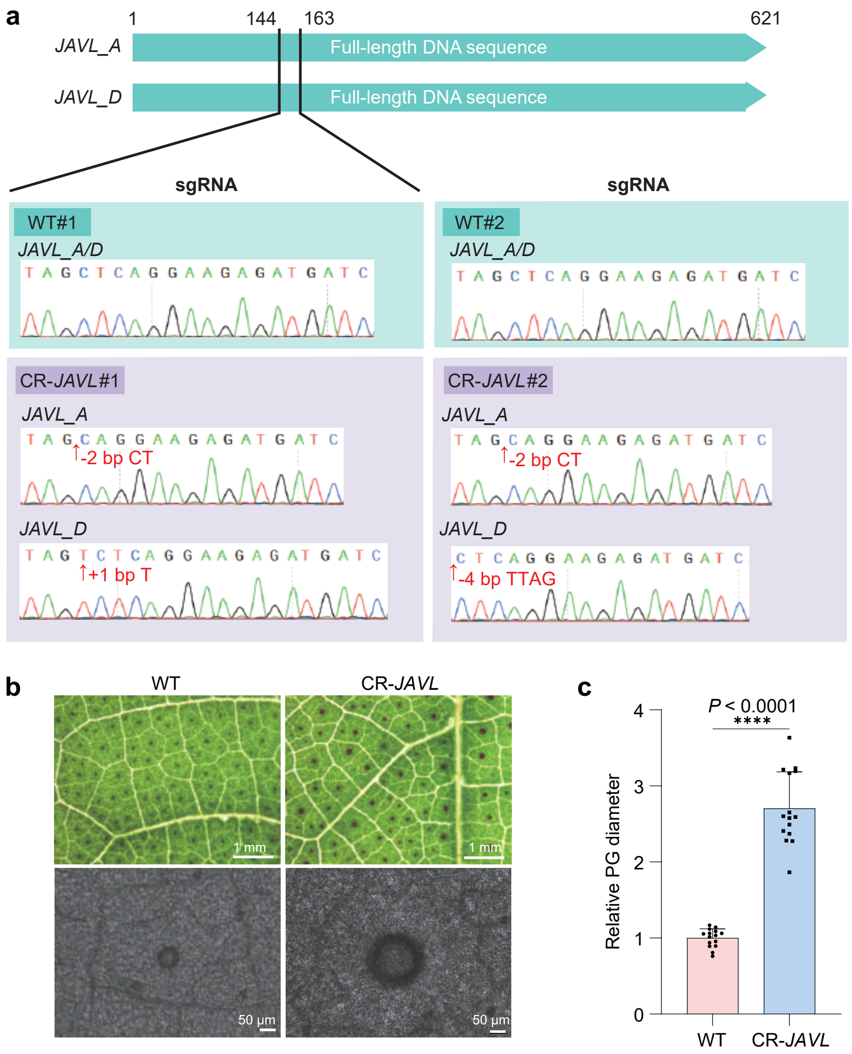

Figure S5. Identification and characterization of *JAVL*-CRISPR plants. a, Genotypes of *JAVL*-CRISPR cotton lines. WT, wild-type plants; CR-*JAVL_A/D*#1/#2, *JAVL*-CRISPR cotton lines. Top: schematic structure of *JAVL_A* and *JAVL_D* gene, with sgRNA indicated between two vertical lines. Bottom: The sequencing results of *JAVL*-knockout lines were shown in the sequencing peak map. **b**, Pigment gland phenotypes comparison between WT and *JAVL*-CRISPR plants. Scale bars are shown in the bottom-right corner of each image. **c**, Relative pigment gland (PG) diameter in leaves of *JAVL*-CRISPR plants (mean ± s.d., n = 15, ****P < 0.0001, Student's t-test).

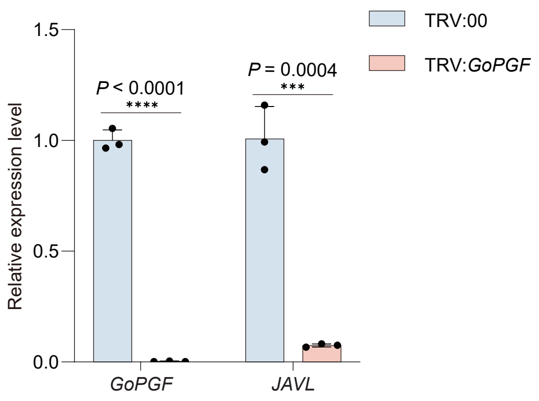

Figure S6. Relative expression of *JAVL* and *GoPGF* in *GoPGF*-silenced cotton leaves by qPCR. Gene expression in control plants (TRV:00) was set as 1. TRV:00, empty vector control cotton plants; TRV:*GoPGF*, *GoPGF*-silenced cotton plants (mean ± s.d., n = 3 ***$P < 0.001$, ****$P < 0.0001$, Student's *t*-test).

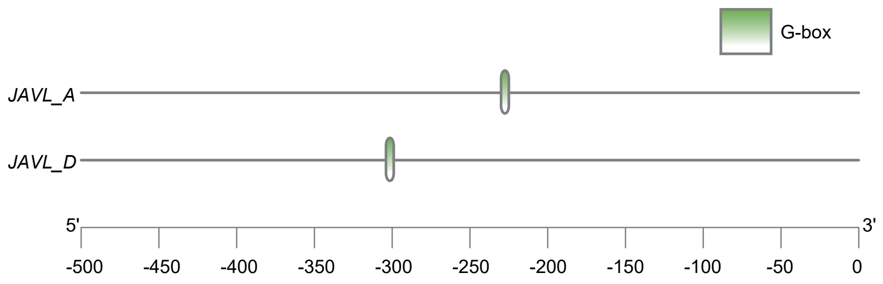

Figure S7. Distribution of the G-box motif in the promoters of *JAVL*.

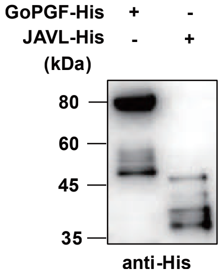

Figure S8. Western blot analysis of GoPGF and JAVL protein. GoPGF-His and JAVL-His were heterologous expressed by *E. coli* BL21(DE3). Molecular weights in kDa were showed on left.

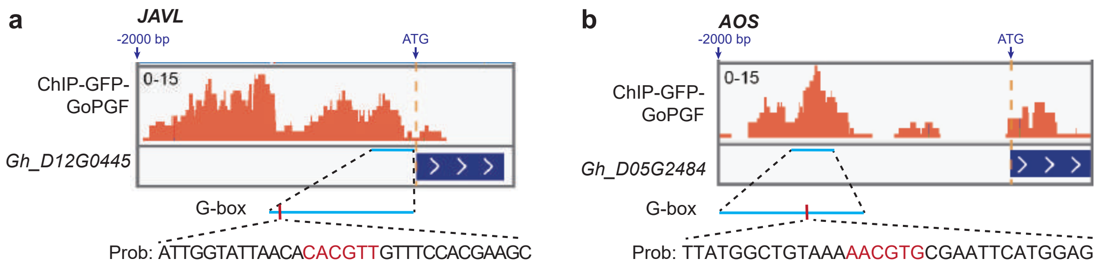

Figure S9. ChIP-seq analysis reveals significant enrichment of GoPGF in the G-box motif in the promoters of *JAVL* (a) and *AOS* (b).

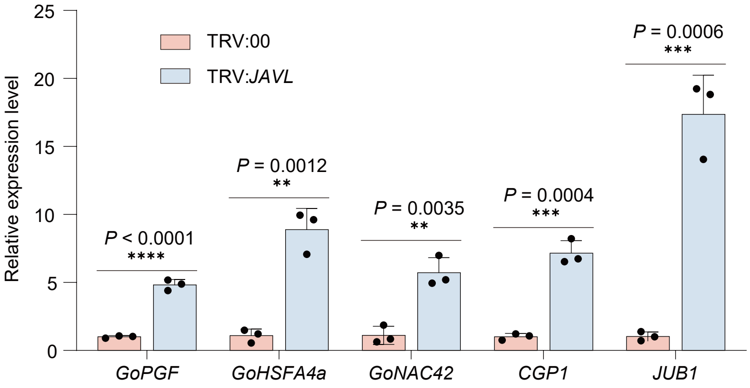

Figure S10. Relative expression of transcription factors specifically expressed in pigment glands in *JAVL*-VIGS cotton leaves by qPCR. Gene expression in control plants (TRV:00) was set as 1. TRV:00, empty vector control cotton plants; TRV:*JAVL*, *JAVL*-VIGS cotton plants; (mean ± s.d., n = 3, $^{**}P < 0.01$, $^{***}P < 0.001$, $^{****}P < 0.0001$, Student's *t*-test).

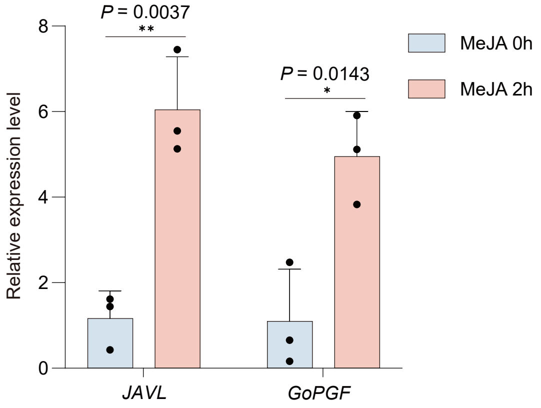

.

Figure S11. Relative expression of *JAVL* and *GoPGF* in cotton seedlings treated by MeJA. Gene expressions of *JAVL* and *GoPGF* in cotyledons were qualified by qPCR after MeJA treatment for 0 h and 2 h. MeJA, methyl jasmonate. Value of MeJA treatment for 0 h was set as 1. (mean ± s.d., n = 3, **P* < 0.05, ***P* < 0.01, Student's *t*-test).

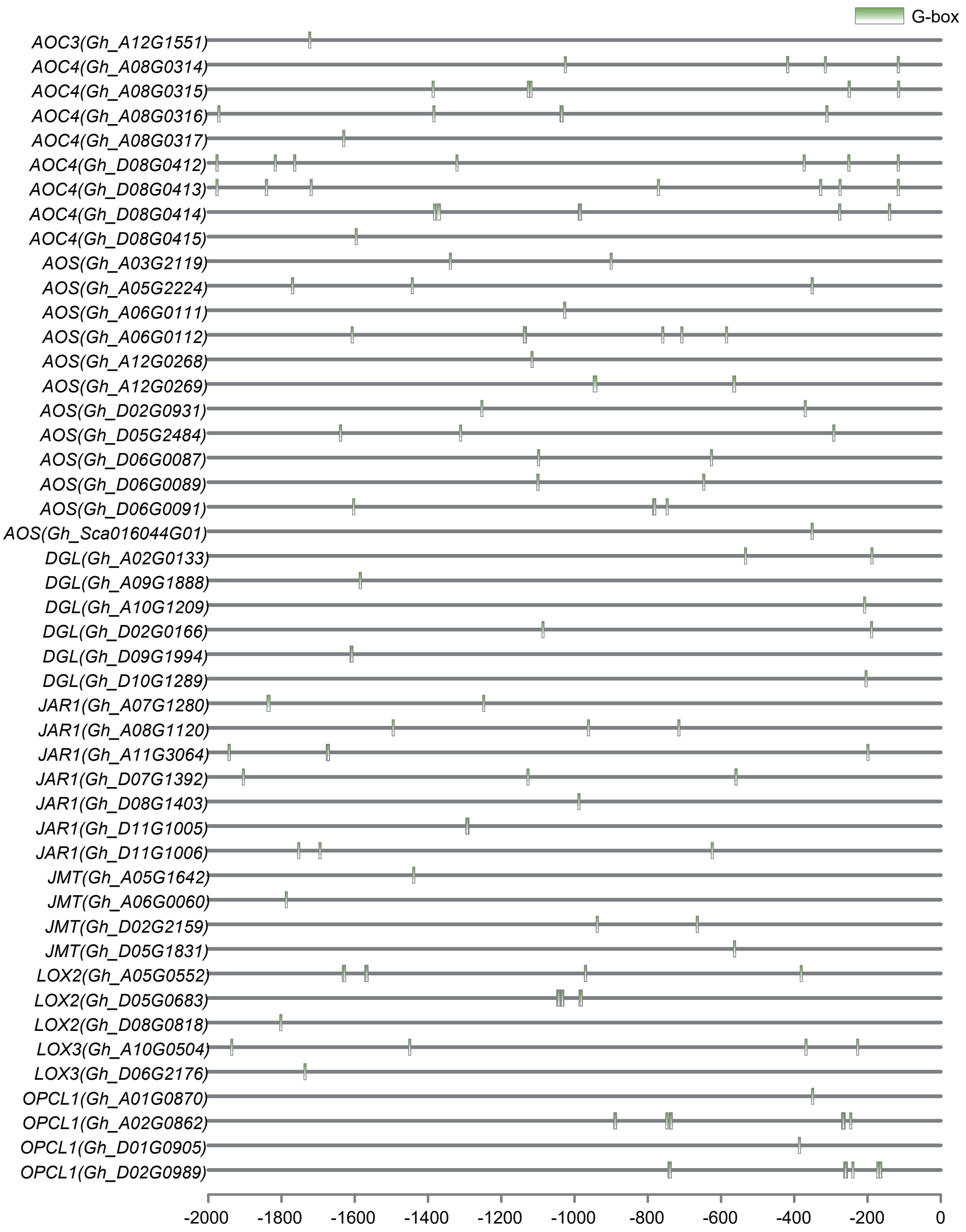

Figure S12. Distribution of G-box motifs in the promoters of jasmonate biosynthesis genes. The upstream 2000 bp sequences of the start codons were extracted as promoters. *AOC*, Allene oxide cyclase; *AOS*, allene oxide synthase; *DGL*, DONGLE; *JAR1*, jasmonoyl amino acid conjugate synthase 1; *JMT*, jasmonic acid carboxyl methyltransferase; *LOX*, lipoxygenase; *OPCL1*, OPC-8:0 CoA Ligase1.

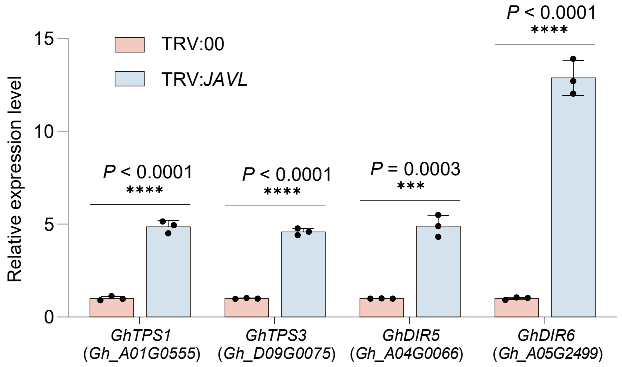

Figure S13. Relative expression of representative gossypol and terpenes biosynthetic genes in *JAVL*-VIGS cotton leaves. Gene expression in control plants (TRV:00) was set as 1. TRV:00, empty vector control cotton plants; TRV:*JAVL*, *JAVL*-VIGS cotton plants (mean ± s.d., n = 3, ****P < 0.0001, ***P < 0.001, Student's t-test).

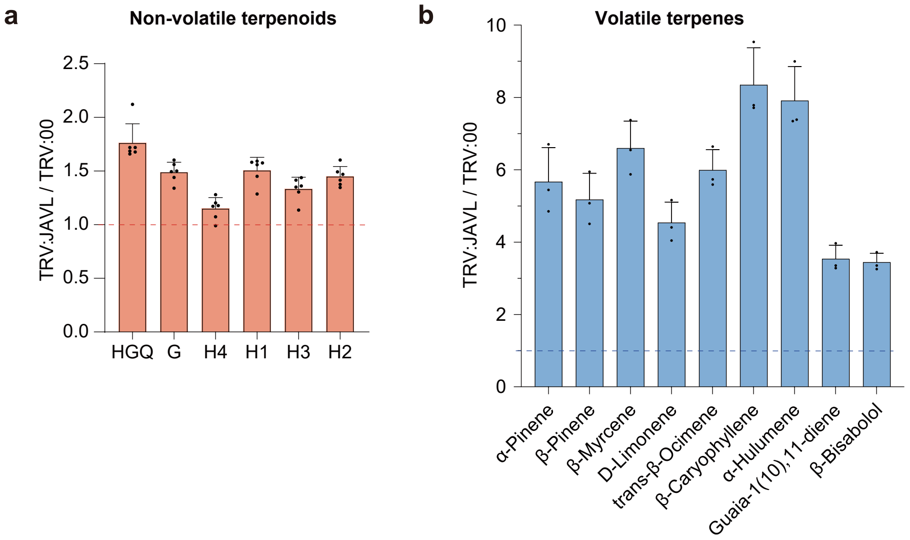

Figure S14. Relative contents of non-volatile terpenoids and volatile terpenes in stems of *JAVL*-VIGS cotton plants. a, Relative contents of non-volatile terpenoids (mean ± s.d., n = 6). HGQ, hemigossypolone; G, gossypol. H1-4 are heliocides. TRV:00, empty vector control cotton plants; TRV:*JAVL*, *JAVL*-VIGS cotton plants. **b**, Relative contents of volatile terpenes (mean ± s.d., n = 3). For figure **a** and **b**, non-volatile terpenoids and volatile terpenes contents in TRV:00 (control) were normalized to 1.

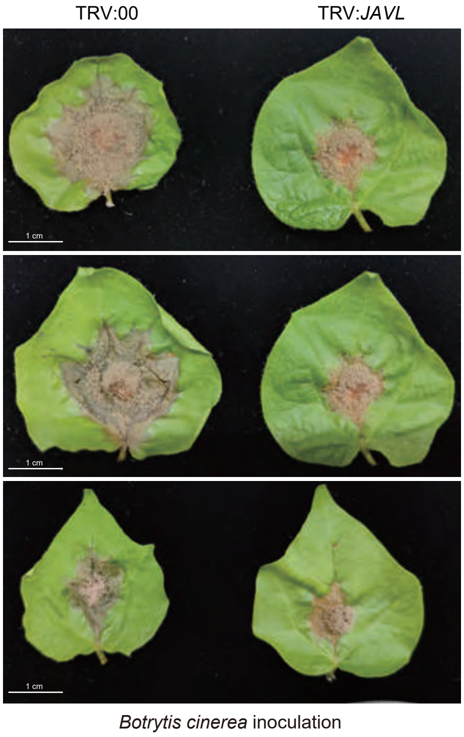

Figure S15. Phenotypes of *JAVL*-VIGS plants inoculation with *Botrytis cinerea*. Lesions were generated by *B. cinerea* in cotton leaves four days after inoculation. TRV:00, empty vector control cotton plants; TRV:-*JAVL*, *JAVL*-VIGS cotton plants. Scale bars, 1 cm.

Table S1. Primers used in this study.

Primer Name	Gene ID	Sequence (5' to 3')	Purpose
JAVL-TRV2-F	*Gh_A12G0442* , *Gh_D12G0445*	GAGTAAGGTTACCGAATTCTCTAGAGAACCTTAGCTCAGGAAGAGATG	Vector construction for VIGS
JAVL-TRV2-R	*Gh_A12G0442* , *Gh_D12G0445*	CGCGTGAGCTCGGTACCGGATCCCCTATTAAACGCCTGCAAAGAAA	Vector construction for VIGS
GoPGF-TRV2-F	*Gh_A12G2172*	AAGGTTACCGAATTCTCTAGATTTGATACACTGGCATGGTGA	Vector construction for VIGS
GoPGF-TRV2-R	*Gh_A12G2172*	CGTGAGCTCGGTACCGGATCCTTGAAGCAGAAAGGCAACGAC	Vector construction for VIGS
JAVL-pCAMBIA1300-YFP-F	*Gh_A12G0442*	CTCGACCCCGGGGGATCCATGAACTCCTCTACAGGAAC	Vector construction for subcellular localization
JAVL-pCAMBIA1300-YFP-R	*Gh_A12G0442*	ATACTAGTTCTAGAGTCGACCAAGAAGTACTTGCCATCAT	Vector construction for subcellular localization
GoPGF-pCAMBIA1300-mCherry-F	*Gh_A12G2172*	AGAGAACACGGGGGACGATGTCTTCCTCTTCTTCGTC	Vector construction for subcellular localization
GoPGF-pCAMBIA1300-mCherry-R	*Gh_A12G2172*	TCGCCCTTGCTCACCATATTCAACGTGCATCTCTGAA	Vector construction for subcellular localization
JAVL-pET32a-F	*Gh_A12G0442*	AGGCCATGGCTGATATCGGATCCATGAACTCCTCTACAGG	Vector construction for protein expression
JAVL-pET32a-R	*Gh_A12G0442*	CCGCAAGCTTGTCGACGGAGCTCCAAGAAGTACTTGCCATCAT	Vector construction for protein expression
GoPGF-pET32a -F	*Gh_A12G2172*	AGGCCATGGCTGATATCGGATCCATGTCTTCCTCTTCTTCG	Vector construction for protein expression
GoPGF-pET32a -R	*Gh_A12G2172*	CCGCAAGCTTGTCGACGGAGCTC CTAATTCAACGTGCATCT	Vector construction for protein expression
JAVL-pEAQ-FLAG-F	*Gh_A12G0442*	CTGCCCAAATTCGCGACCGGTATGAACTCCTCTACAGGAA	Vector construction for protein expression
JAVL-pEAQ-FLAG-R	*Gh_A12G0442*	TCGTCGTCATCCTTGTAATCGTCGACCAAGAAGTACTTGCCATCAT	Vector construction for protein expression
GoPGF-pEAQ-FLAG-F	*Gh_A12G2172*	CTGCCCAAATTCGCGACCGGTATGTCTTCCTCTTCTTCGT	Vector construction for protein expression
GoPGF-pEAQ-FLAG-R	*Gh_A12G2172*	TCGTCGTCATCCTTGTAATCGTCGACATTCAACGTGCATCTCTGAA	Vector construction for protein expression
pJAVL-phis2.1-F	*Gh_A12G0442*	GACTCACTATAGGGCGAATTC GGTGAGGGTGGATGCAGATT	Vector construction for Y2H
pJAVL-phis2.1-R	*Gh_A12G0442*	ATAATGCCAGGAATTACTAGT GGAAAGGAATAATATC	Vector construction for Y2H
pAOS-phis2.1-F	*Gh_D05G2484*	GACTCACTATAGGGCGAATTCTCTCCAATACCGAATGTCA	Vector construction for Y2H
pAOS-phis2.1-R	*Gh_D05G2484*	ATAATGCCAGGAATTACTAGTTTGAATAGAGTAACAATGAGCTTC	Vector construction for Y2H
JAVL-pGBKT7-F	*Gh_A12G0442*	GAGGAGGACCTGCATATGAACTCCTCTACAGGAACCTC	Vector construction for Y2H
JAVL-pGBKT7-R	*Gh_A12G0442*	TTATGCGGCCGCTGCTTACAAGAAGTACTTGCCATCATTT	Vector construction for Y2H
GoPGF-pGADT7-F	*Gh_A12G2172*	CCAGATTACGCTCATATGTCTTCCTCTTCTTCGTCTTCT	Vector construction for Y2H
GoPGF-pGADT7-R	*Gh_A12G2172*	CTACGATTCATCTGCCTAATTCAACGTGCATCTCTGAAGG	Vector construction for Y2H
JAVL-JW771-F	*Gh_A12G0442*	TCCCGGGGCGGTACCATGAACTCCTCTACAGGAACCTC	Vector construction for SLC
JAVL-JW771-R	*Gh_A12G0442*	GCTCTGCAGGTCGACCAAGAAGTACTTGCCATCATTT	Vector construction for SLC
GoPGF-JW772-F	*Gh_A12G2172*	TCCCGGGGCGGTACCATGTCTTCCTCTTCTTCGTCTTCT	Vector construction for SLC
GoPGF-JW772-R	*Gh_A12G2172*	GCTCTGCAGGTCGACCTAATTCAACGTGCATCTCTGAAGG	Vector construction for SLC
pJAVL-0800-F	*Gh_A12G0442*	TATAGGGCGAATTGGGTACCGGTGAGGGTGGATGCAGATT	Vector construction for dual-LUC
pJAVL-0800-R	*Gh_A12G0442*	AAGGGTCTTGCGGGATCCGGAAAGGAATAATATCAATTCAC	Vector construction for dual-LUC
pGoPGF-0800-F	*Gh_A12G2172*	TATAGGGCGAATTGGGTACCTTCTTTTGATGGGGGTCAGACA	Vector construction for dual-LUC
pGoPGF-0800-R	*Gh_A12G2172*	AAGGGTCTTGCGGGATCCTATTGAATATGATAGTGTGTACTACCG	Vector construction for dual-LUC
pAOS-0800-F	*Gh_D05G2484*	TATAGGGCGAATTGGGTACCATCTCCAATACCGAATGTCA	Vector construction for dual-LUC
pAOS-0800-R	*Gh_D05G2484*	AAGGGTCTTGCGGGATCCTTGAATAGAGTAACAATGAGCTTC	Vector construction for dual-LUC
GhUB7-qPCR-F	*Gh_A11G0969*	GAAGGCATTCCACCTGACCAAC	qPCR primer for biomass
GhUB7-qPCR-R	*Gh_A11G0969*	CTTGACCTTCTTCTTCTTGTGCTTG	qPCR primer for biomass
BcActin-qPCR-F	*BCIN_16g02020*	ATGGTATTATGATTGGTATGGGT	qPCR primer for biomass
BcActin-qPCR-R	*BCIN_16g02020*	GGGAGAGGA CGGCTTGAATAGAGAC	qPCR primer for biomass
GhHis-qPCR-F	*Gh_D03G0370*	GGCATACCTTGTGGGTCTTTTTGA	qPCR primer for gene expression
GhHis-qPCR-R	*Gh_D03G0370*	CTACCACTACCATCATGGC	qPCR primer for gene expression
JAVL-qPCR-F	*Gh_A12G0442* , *Gh_D12G0445*	TCTGATGTTGATGCCACTAT	qPCR primer for gene expression
JAVL-qPCR-R	*Gh_A12G0442* , *Gh_D12G0445*	GCCTCTTCTGTTGTTGGT	qPCR primer for gene expression
GoPGF-qPCR-F	*Gh_A12G2172*	TCTGATGGGGATTTCGCCTCTG	qPCR primer for gene expression
GoPGF-qPCR-R	*Gh_A12G2172*	GGATTTGGTAGTTTGGTTTTCG	qPCR primer for gene expression
GoHSFA4a-qPCR-F	*Gh_A05G3255*	AGCCAATCCATAGCCATTC	qPCR primer for gene expression
GoHSFA4a-qPCR-R	*Gh_A05G3255*	CTCCGATAACAGCCTTACTT	qPCR primer for gene expression
GoNAC42-qPCR-F	*Gh_A06G1947*	GACTGGGATCGACAAGCCAA	qPCR primer for gene expression
GoNAC42-qPCR-R	*Gh_A06G1947*	TGCATACTGTCCAAACTTCAGC	qPCR primer for gene expression
CGP1-qPCR-F	*Gh_A07G0703*	TTGCTGCTGATGAAGTTGA	qPCR primer for gene expression
CGP1-qPCR-R	*Gh_A07G0703*	GGCTTGATAACCTTAGTATTGG	qPCR primer for gene expression
JUB1-qPCR-F	*Gh_A01G0267*	TCATCATAACGACGAGGAG	qPCR primer for gene expression
JUB1-qPCR-R	*Gh_A01G0267*	GCAGCACTACTCATTCTATC	qPCR primer for gene expression
AOS-qPCR-F	*Gh_D05G2484*	GTCTTCTCGGCGGATTAG	qPCR primer for gene expression
AOS-qPCR-R	*Gh_D05G2484*	TGAATGGACCAACAATAGGA	qPCR primer for gene expression
AOC4-qPCR-F	*Gh_D08G0413*	CCAGGCTATTCCATCTGATAA	qPCR primer for gene expression
AOC4-qPCR-R	*Gh_D08G0413*	ACCGTAGTCTCCGAAGTAA	qPCR primer for gene expression
JAR1-qPCR-F	*Gh_A08G1120*	TTCGCAATTCACAGTTCAAG	qPCR primer for gene expression
JAR1-qPCR-R	*Gh_A08G1120*	GAGATGGCAGTACAAGGATT	qPCR primer for gene expression
LOX3-qPCR-F	*Gh_D06G2176*	TGGAATACGATGCGATGG	qPCR primer for gene expression
LOX3-qPCR-R	*Gh_D06G2176*	TTGTTGGTGACGAGGATAG	qPCR primer for gene expression
GhTPS1-qPCR-F	*Gh_A01G0555*	ACCGATGGTAGAAGAGTA	qPCR primer for gene expression
GhTPS1-qPCR-R	*Gh_A01G0555*	CTTAGGGTTGTTAGATGC	qPCR primer for gene expression
GhTPS3-qPCR-F	*Gh_D09G0075*	CAGGCTCAGTGGAAACGC	qPCR primer for gene expression
GhTPS3-qPCR-R	*Gh_D09G0075*	AGTTTGCTCGCTCGTTCC	qPCR primer for gene expression
GhDIR5-qPCR-F	*Gh_A04G0066*	TGCCATTTGCCACCCTAGTT	qPCR primer for gene expression
GhDIR5-qPCR-R	*Gh_A04G0066*	CGCTTAGAGAGCTGCCGTTA	qPCR primer for gene expression
GhDIR6-qPCR-F	*Gh_A05G2499*	TCCCTCCTAGCCGGATCAAA	qPCR primer for gene expression
GhDIR6-qPCR-R	*Gh_A05G2499*	CGCTTAGAGAGCTGCCGTTA	qPCR primer for gene expression
JAVL-probe-cy5	*Gh_D12G0445*	ATTGGTATTAACACACGTTGTTTCCACGAAGC	EMSA probe
JAVL-probe-mutant	*Gh_D12G0445*	ATTGGTATTAACAACATGGGTTTCCACGAAGC	EMSA probe
AOS-probe-cy5	*Gh_D05G2484*	TTATGGCTGTAAAAACGTGCGAATTCATGGAG	EMSA probe
AOS-probe-mutant	*Gh_D05G2484*	TTATGGCTGTAAACCATGTCGAATTCATGGAG	EMSA probe
GoPGF-probe-cy5	*Gh_A12G2172*	AACAGGAGATTTCCACGTGATAATGATGTCAA	EMSA probe
GoPGF-probe-mutant	*Gh_A12G2172*	AACAGGAGATTTCACATGTATAATGATGTCAA	EMSA probe
